# Phylogeographic analyses reveal recent dispersal and multiple *Wolbachia* infections of the bright-eyed ringlet *Erebia oeme* within the European mountain systems

**DOI:** 10.1038/s41598-024-84551-5

**Published:** 2025-01-14

**Authors:** Valentine Mewis, Martin Wendt, Thomas Schmitt

**Affiliations:** 1 Senckenberg Deutsches Entomologisches Institut, Systematik und Biogeographie, Eberswalder Str. 90, 15374 Müncheberg, Germany; 2 Leibniz-Zentrum für Agrarlandschaftsforschung (ZALF) e.V, Eberswalder Str. 84, 15374 Müncheberg, Germany; 3 Entomology and Biogeography, Institute of Biochemistry and Biology, Faculty of Science, University of Potsdam, 14476 Potsdam, Germany

**Keywords:** Biogeography, Pleistocene, Range dynamics, Glacial refugia, Butterflies, Mountain species, Biodiversity, Biogeography, Conservation biology, Population genetics, Entomology

## Abstract

The genus *Erebia* comprises numerous species in Europe. Due to preference of cold environments, most species have disjunct distributions in the European mountain systems. However, their biogeographical patterns may differ significantly. The Bright-eyed ringlet *Erebia oeme* is widespread in high-altitude grasslands of the European high mountains, hence showing a disjunct distribution pattern. Over its distribution, *E. oeme* shows high morphological variability indicating pronounced intraspecific differentiation. We analysed two mitochondrial (COI, Cytb) and eight nuclear markers as well as the *Wolbachia* surface protein-coding gene (WSP). A total of four lineages were identified: two Balkan lineages, one Slovenian lineage as well as one lineage containing all other individuals (Alps, Massif Central, Pyrenees). COI data only indicate a fifth lineage in the southern Carpathians. The region of origin of *E. oeme* is most likely the western Balkans. From here, *E. oeme* spread to the eastern Balkan area and further to the southern Carpathians as well as to Slovenia, from where it rapidly crossed the Alps to Massif Central and Pyrenees. *Wolbachia* was found to be highly prevalent with multiple strains, partly within one population. However, recent *Wolbachia* impact on the mitochondrial DNA and consequently influence on the intraspecific genetic structure was not detected.

**Supplementary Information:**

The online version contains supplementary material available at 10.1038/s41598-024-84551-5.

## Introduction

Flora and fauna of the western Palaearctic are classified into different biogeographic types according to their distribution patterns and evolutionary histories. One main type comprises species with a preference to cold environments. These cold-adapted species are assumed to possess wide distributions due to range expansion during cold periods and limited distributions in combination with vicariance events during warm periods^1,2^. Accordingly, their today’s distributions often extend disjunctively within or across mountain ranges and, partly additional, at higher northern latitudes of Eurasia^3^. However, the biogeographical distribution patterns of these species are often highly diverse^3^. For instance, species with an alpine disjunction are distributed in (parts of) one or more mountain ranges of the western Palaearctic north of the Adamović line^3,4^. In this context, the Alps are of great importance, as they not only have a high abundance of endemic species, but also are frequently colonised by more widespread, disjunctly distributed species^3,4^. In addition, genetic differentiation within the Alps is typically pronounced due to the assumed survival of alpine species in multiple refugia around or even within the Alps during glacial periods and the following range modifications of the distinct groups during interglacial periods^5–7^. Similarly pronounced genetic differentiation within alpine species also exist at the Balkan Peninsula, especially between its eastern and western parts^8,9^. In other European mountain ranges, such as the Pyrenees or the Carpathians, genetic differentiation is generally less pronounced than in the Alps or at the Balkan Peninsula^4^. However, within geographically distinct mountain ranges, identical or closely related genetic lineages of one species can occur due to biogeographical links between them^10^. In this context, the Alps are also of great significance because biogeographical connections to almost all other western Palearctic mountain ranges exist^6,11,12^. Due to this complexity of their distribution, alpine species often exhibit strong population genetic structures^4,7^.

**Fig. 1:**
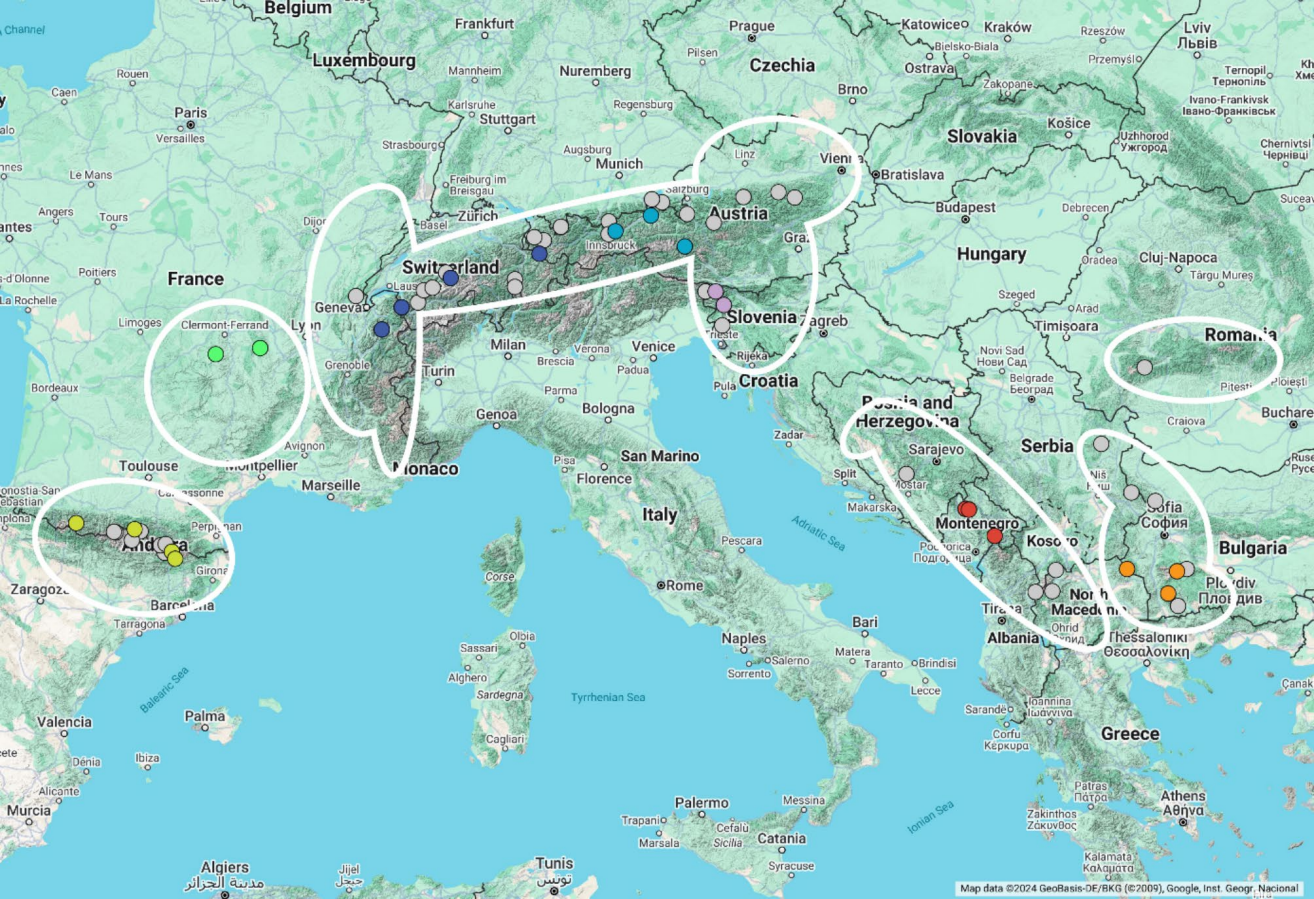
Map of the sampling localities (points) and distribution area (white area) of *Erebia oeme*. Chartreuse—Pyrenees, Green—Massif Central, Blue—Central and Western Alps, Light blue—Eastern Alps, Lilac—Julian Alps, Red—Western Balkan Peninsula, Orange—Eastern Balkan Peninsula, Grey—GenBank sequences. Map created using Google My Maps (https://www.google.com/mymaps/) and further modified in PowerPoint (Microsoft PowerPoint for Microsoft 365 MSO, Version 2410 Build 16.0.18129.20158, 64-bit). Map data sources include: GeoBasis-DE/BKG 2024, Google, Instituto Geográfico Nacional. Additional distribution data adapted from^14^.

A prime example of a taxon containing a large number of cold-adapted species, including numerous alpine elements, is the species-rich butterfly genus *Erebia*^3,13^. One species belonging to this genus is the Bright-eyed ringlet *Erebia oeme* (Hübner 1804). This mountain butterfly species is endemic to Europe and exhibits an alpine disjunction with a widespread distribution in the mountain systems of Central and South Europe (Fig. 1)^3,14,15^. In contrast to other alpine elements, *E. oeme* does not occur at the highest altitudes, but can be found at altitudes as low as 600 m asl., mainly in the range 1200–2600 m asl^14,16^. There, the Bright-eyed ringlet inhabits mesic to wet grassland and mountain fields^3,15,17^.

Species of the genus *Erebia*, including *E. oeme*, generally possess a relatively high prevalence for *Wolbachia* infections^7,18^. *Wolbachia* is an intracellular, maternally inherited, parasitic bacterium of invertebrates^19^. The presence and effects of *Wolbachia* can have consequences on the evolution of the hosts. For example, gene flow between host populations and closely related taxa may be reduced due to *Wolbachia*^20,21^. This in turn may result in reproductive isolation or diversification of host populations and in the long run even speciation^20,22,23^. The impact of *Wolbachia* is particularly evident in the mitochondrial DNA: the genetic diversity, effective population size and polymorphism of mitochondrial genes as well as the mitochondrial divergence between species can be partly significantly reduced^24–27^.

*E. oeme* offers a quite high morphological variability across its distribution area suggesting the existence of enhanced intraspecific differentiation. Despite this, research attention so far has been relatively limited in the case of *E. oeme*. In previous studies involving this species, the taxon has usually been included but not focused on [e.g., ^28–30^]. An exception is the study conducted by Dinca et al. (2010), which analysed *E. oeme* from the Carpathian both morphologically and genetically^31^. However, detailed genetic and phylogeographic studies are currently lacking. In order to gain a deeper insight into the phylogeny and biogeography of *E. oeme*, the following research questions will be addressed:

(I) Can the intraspecific differentiation assumed based on morphological features also be observed at the genetic level?
(II) Where does *E. oeme* originate from and when and how did the species disperse from this area of origin?
(III) Is *E. oeme* infected with the parasitic bacterium *Wolbachia*?
(IV) If so, is *E. oeme* infested with different *Wolbachia* strains and what are the phylogeographic consequences of these *Wolbachia* infections?

In order to investigate the phylogeographic structure and biogeographic patterns of this species, we analysed individuals from most of its distribution area using two mitochondrial and several nuclear markers. In addition, due to the high prevalence in *Erebia* species and the potential impact on mitochondrial markers, all individuals were tested for *Wolbachia* infection.

## Materials and methods

### Sampling and specimens

In this study, 166 specimens representing 20 populations of *E. oeme* (**Table ****S1**) were included. Specimens were collected in the Pyrenees (4 localities), Massif Central (2), Alps (9) and the Balkan Peninsula (5) in 2005‒2014 (Fig. 1). The samples were directly frozen and stored in liquid nitrogen or stored in 96% ethanol and afterwards stored at -20 or -80 °C, respectively. In addition, a total of 56 barcode sequences from GenBank of *E. oeme* from the Pyrenees (14 sequences), Jura (2), Alps, (23), Balkan Peninsula (11) and southern Carpathians (6) were included (**Table S2**).

### Molecular methods

At first, DNA was extracted from two legs of each *E. oeme* sample using the E.Z.N.A. Tissue DNA Kit (Omega Bio-tek) following the “DNA Extraction and Purification from Tissue” protocol with minor modifications (**Table S3**). Next, two mitochondrial gene fragments, cytochrome c oxidase subunit I (COI, 1223 bp) and Cytochrome b (Cytb, 586 bp), as well as the *Wolbachia* surface protein-coding gene (WSP, 549 bp) were amplified and sequenced for all individuals. The total volume of each PCR reaction of COI was 25 µL containing 12.5 µL Qiagen Master Mix, 1.5 µL primer, 1 µL DNA and 10 µL water. The amplification was done with the primers LepF1 (5’-ATTCAACCAATCATAAAGATATTGG-3’)^32^, Nancy (5’-CCCGGTAAAATTAAAATATAACTTC-3), Tonya (5’-GAAGTTTATATTTTAATTTTACCGGG 3’) and Hobbes (5’-AAATGTTGNGGRAAAAATGTTA-3’)^33^ and the following PCR protocol: 5’ 95°C, 38 × (30’’ 95°C, 90” 49°C, 90” 72°C) 30’ 68°C. For Cytb, each PCR volume was 15 µL. This contained 7.5 µL of Qiagen Master Mix, 1 µL of primers, 1–2 µL of DNA and made up with water. Cytb was amplified using the primers REVCB2H (5’-TGAGGACAAATATCATTTTGAGGW-3) and REVCBJ (5’-ACTGGTCGAGCTCCAATTCATGT-3’)^34^ and the following PCR program: 5’ 95°C, 40 × (30’’ 95°C, 90” 50°C, 60” 72°C) 30’ 68°C. For WSP, PCR volume was 15 µL, containing 7.5 µL of Qiagen Master Mix, 1.5 µL of primers, 1–2 µL of DNA and made up with water. The amplification was done with wsp81F (5’-TGGTCCAATAAGTGATGAAGAAAC-3’) and wsp691R (5’-AAAAATTAAACGCTACTCCA-3’)^35^ as primers and the following PCR program: 5’ 95°C, 40 × (30’’ 95°C, 90” 54°C, 60” 72°C) 30’ 68 °C. PCR success was verified by electrophoresis using 1.5% agarose gels. Afterwards, PCR products were purified and prepared for sequencing using the corresponding PCR primers. Sanger sequencing of sense and antisense strands was conducted by Macrogen Europe (Amsterdam, The Netherlands).

We tested the nuclear markers carbamoyl-phosphate synthetase 2, aspartate transcarbamylase, dihydroorotase (CAD, 856 bp), eukaryotic translation elongation factor 1 alpha (EF1α, 957 bp), glyceraldehyde-3-phosphate dehydrogenase (GAPDH, 693 bp), isocitrate dehydrogenase (IDH, 723 bp), cytosolic malate dehydrogenase (MDH, 739 bp), Nedd2-like caspase (NC, 607 bp), ribosomal protein S5 (RPS5, 614 bp) and wingless (wg, 425 bp). These genes were only sequenced for a fraction of the individuals. For detailed PCR protocols of the nuclear markers see **Table S4** in the supplementary.

### Statistical analyses

Sequences were edited with GENEIOUS v9.1.8^36^ and implemented and manually aligned in BIOEDIT v7.2.5^37^. Analyses were performed in R v4.3.3^38^, BEAST v2.6.7^39^ and RASP v4.3^40^. The used datasets included on the one hand the sequences of COI and Cytb from 166 specimens (main dataset) and on the other hand 222 barcode sequences (i.e. subunit 1 of COI), consisting of the 166 specimens and additional 56 GenBank sequences (barcode dataset).

For phylogenetic analyses of the main dataset, first the number of haplotypes, haplotype and nucleotide diversity as well as segregating sites were calculated for the used markers and the genetic clusters of *E. oeme* using the R packages “pegas”^41^ and “ape”^42^. Pairwise genetic distances were calculated using TrN as the best-fitting substitution model using “ape”^42^ and “phangorn”^43,44^ and visualised in a heatmap with dendrogram. Mismatch analysis and tests of the neutral mutation hypothesis with Tajima’s D for the corresponding genetic clusters were performed using “pegas”^41^ and “adegenet”^45,46^. Furthermore, principal component analysis (PCA) and non-metric multidimensional scaling (NMDS) were done using the packages “stats”^38^ and “vegan”^47^. TCS haplotype networks were constructed using “pegas”^41^.

Bayesian trees of the haplotypes of *E. oeme* were constructed in BEAST. The substitution model for each gene fragment was selected based on the lowest Akaike Information Criterion (AIC) calculated in JMODELTEST v2.1.9^48,49^. The used model was HKY + G + I for COI and HKY + G for Cytb. For molecular clock analysis, the relaxed clock log normal for each mitochondrial gene fragment was used with a clock rate of 0.0177^50^. For the tree priors, the Yule model was used for all genes. MCMC was set to 30 million generations saving trees every 3,000 generations. Convergence and stationarity were checked with TRACER v1.7.2^51^. The BEAST package LOGCOMBINER v2.6.7. was used to combine separate tree-files obtained for different markers. A consensus tree with a burn-in percentage of 10 and a posterior probability limit of 0.5 was computed using TREEANNOTATOR v2.6.7 of the BEAST package. The consensus tree was visualized in FIGTREE v.1.4.4^52^. The node ages were calculated by BEAST using the same settings described for the Bayesian trees.

In RASP, a Statistical Dispersal-Vicariance Analysis (S-DIVA) was performed using the Bayesian trees from BEAST as input data. We applied default settings.

For the barcode dataset, a haplotype network was generated in R using the same settings described for the main dataset. In addition, a Bayesian tree of the haplotypes was constructed in BEAST using HKY + G as the substitution model with the lowest AIC. The other settings were identical to those for the main dataset.

The *Wolbachia* infection status of each sample was determined by electrophoresis (**Table S7**). The corresponding *Wolbachia* strain was manually identified in BIOEDIT v7.2.5^37^. Pairwise genetic distances of the detected *Wolbachia* strains were calculated with TrN as the best-fitting substitution model using “ape”^42^ and “phangorn”^43,44^. The Maximum likelihood phylogeny with bootstrap values of all detected strains was performed using TVM + I as best-suited substitution model and the “phangorn” package^43,44^.

## Results

### Phylogeographic analyses of *Erebia oeme*

The tested nuclear markers all showed a variability of less than 1% (Table 1), with MDH (7 variable sites; 0.95% of all sites), CAD (8; 0.93%) and RPS5 (5; 0.81%) being most variable. However, these markers proved to be unsuitable for further analyses, among others due to the lack of variability among the different mountain ranges.

**Table 1:** Variability of the tested nuclear markers of *Erebia oeme.*

Marker system	Molecular marker	Number of sequences	Variable positions
Nuclear	CAD	17	8 (0.93%)
	EF1α	17	5 (0.52%)
	GAPDH	17	1 (0.14%)
	IDH	17	2 (0.28%)
	MDH	15	7 (0.95%)
	NC	16	1 (0.16%)
	RPS5	6	5 (0.81%)
	WG	15	1 (0.24%)

The mitochondrial dataset had a total intraspecific variability of 3.13% representing 39 haplotypes, as well as a haplotype and nucleotide diversity of 0.942 and 0.00321, respectively, with 54 segregating sites (Table 2).

**Table 2:** Genetic diversity parameters and variability of the used mitochondrial markers of *Erebia oeme*.

Marker/marker system	Variability	*n*	*h*	*Pi*	*S*
COI	35 (2.86%)	30	0.859	0.00285	34
Cytb	21 (3.69%)	22	0.838	0.00400	20
Mitochondrial (total)	56 (3.13%)	39	0.942	0.00321	54

*n* number of haplotypes, *h* haplotype diversity, *Pi* nucleotide diversity, *S* Segregating sites.

We obtained 21 haplotypes in the Alps, Massif Central and Pyrenees (for the sake of simplicity hereinafter referred to as non-Balkan group) and 15 in the samples from the Balkan Peninsula, with a haplotype and nucleotide diversity of 0.894 and 0.00129 as well as 0.900 and 0.00452, respectively (Table 3). The Julian Alps (Slovenia) showed three haplotypes and a haplotype and nucleotide diversity of 0.833 and 0.00093. The number of segregating sites per mountain region ranges from 32 in the Balkan region to three in the Julian Alps (Slovenia) and the Massif Central.

**Table 3:** Genetic diversity parameter of the main dataset (COI and Cytb) of *Erebia oeme* from the different mountain ranges and regions.

Mountain range	Region	*N*	*n*	*h*	*Pi*	*S*
Pyrenees	Pyrenees	40	5	0.627	0.00047	5
Massif Central	Massif Central	20	5	0.700	0.00058	3
Alps	Alps (total)	58	16	0.912	0.00169	20
	Alps without Slovenia	54	13	0.899	0.00149	15
	Julian Alps (Slovenia)	4	3	0.833	0.00093	3
	Central and Western Alps	33	8	0.831	0.00160	11
	Eastern Alps	25	8	0.810	0.00140	10
	Eastern Alps without Slovenia	21	5	0.733	0.00090	5
Non-Balkan	Pyrenees, Massif Central, Alps without Slovenia	114	21	0.894	0.00129	23
Balkan Peninsula	Balkan Peninsula	48	15	0.900	0.00452	32
	Western Balkan Peninsula	17	9	0.846	0.00338	20
	Eastern Balkan Peninsula	31	6	0.802	0.00345	15
Total	All	166	39	0.942	0.00321	54

*N* Number of samples included in the cluster, *n* number of haplotypes, *h* haplotype diversity, *Pi* nucleotide diversity, *S* Segregating sites.

**Fig. 2:**
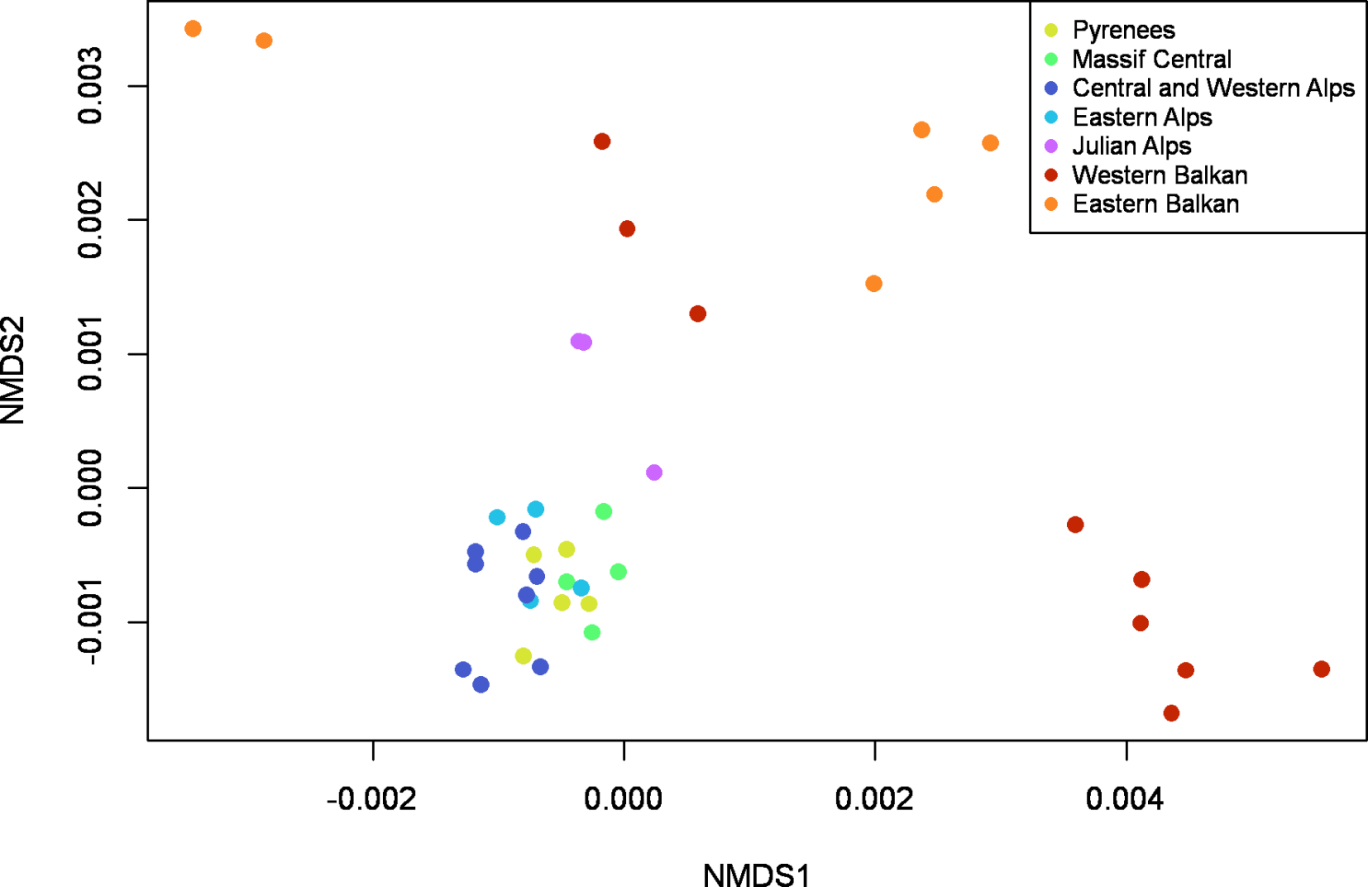
Non-metric multidimensional scaling (NMDS) of *Erebia oeme* from the different mountain regions. The corresponding colour codes to each region are given in the legend.

Pairwise genetic distances of the *E. oeme* samples revealed two main groups, with samples from the Balkan region and Sija (Slovenia) in one group as well as samples from the Alps (including the second population from Slovenia), Massif Central and Pyrenees in the second group (**Figure ****S1**). The largest genetic distance between these two groups was about 0.010. Both main groups showed several subgroups, strongly developed in the Balkan group, weakly in the non-Balkan group. Non-metric multidimensional scaling (NMDS) analysis (Fig. 2) identified the samples from the Alps, Massif Central and Pyrenees as one coherent group; the Balkan group was not identified as one closed cluster, but rather as four groups clustering next to each other; the Julian Alps represented a link between the non-Balkan and Balkan specimens. Principal component analysis (PCA) (**Figure S2**,** S3**) revealed similar results and recognised a strongly substructured group consisting of the Balkan samples and a more coherent group containing the non-Balkan samples. In contrast to the tree approach based on genetic distances, the samples from the Julian Alps (Slovenia) were not clearly assigned to one of the two groups in ordinations but were located mid-distance between these two main groups.

**Fig. 3:**
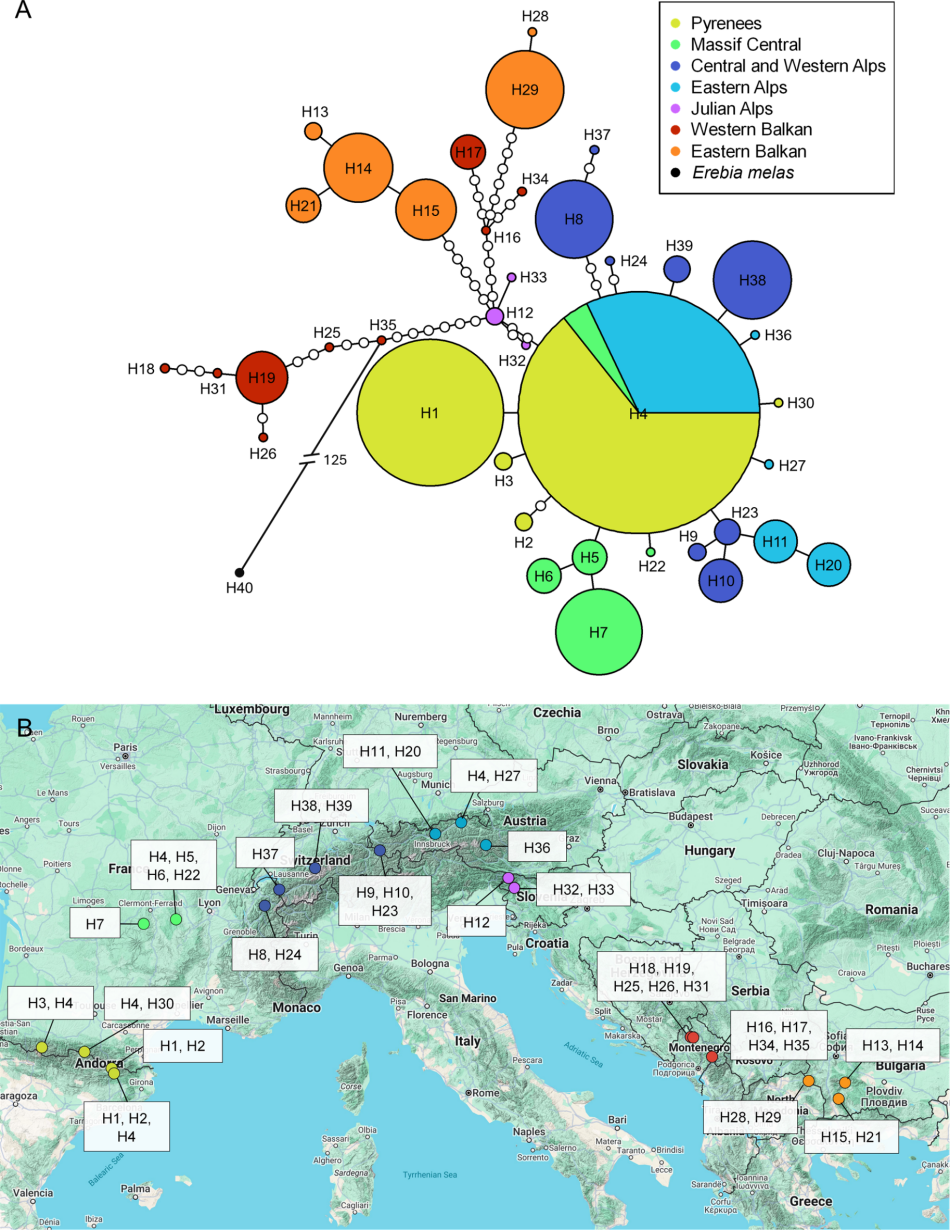
Haplotypes based on the mitochondrial markers COI and Cytb of *Erebia oeme*. (**A**) TCS haplotype network. The small white circles on the links represent missing haplotypes. The corresponding colour codes to each region are given in the legend. (**B**) Geographic distribution of the haplotypes. Map created using Google My Maps (https://www.google.com/mymaps/) and further modified in PowerPoint (Microsoft PowerPoint for Microsoft 365 MSO, Version 2410 Build 16.0.18129.20158, 64-bit). Map data sources include: GeoBasis-DE/BKG 2024, Google, Instituto Geográfico Nacional.

Similar observations were made in the haplotype network (Fig. 3) which showed one group consisting of 15 haplotypes from the mountains of the eastern and western Balkan Peninsula and one non-Balkan group containing 21 haplotypes. In this case, too, the Slovenian samples represent the link between the Balkan and non-Balkan group. The Balkan group showed a branched structure with no dominant haplotype and more missing haplotypes between the detected individual haplotypes than in the non-Balkan group. In contrast, the non-Balkan group presented a star-like structure with the dominant H4, which included samples from the Pyrenees, Massif Central and Eastern Alps. The derived haplotypes were separated from this main haplotype by one to a maximum of five mutational steps.

**Fig. 4:**
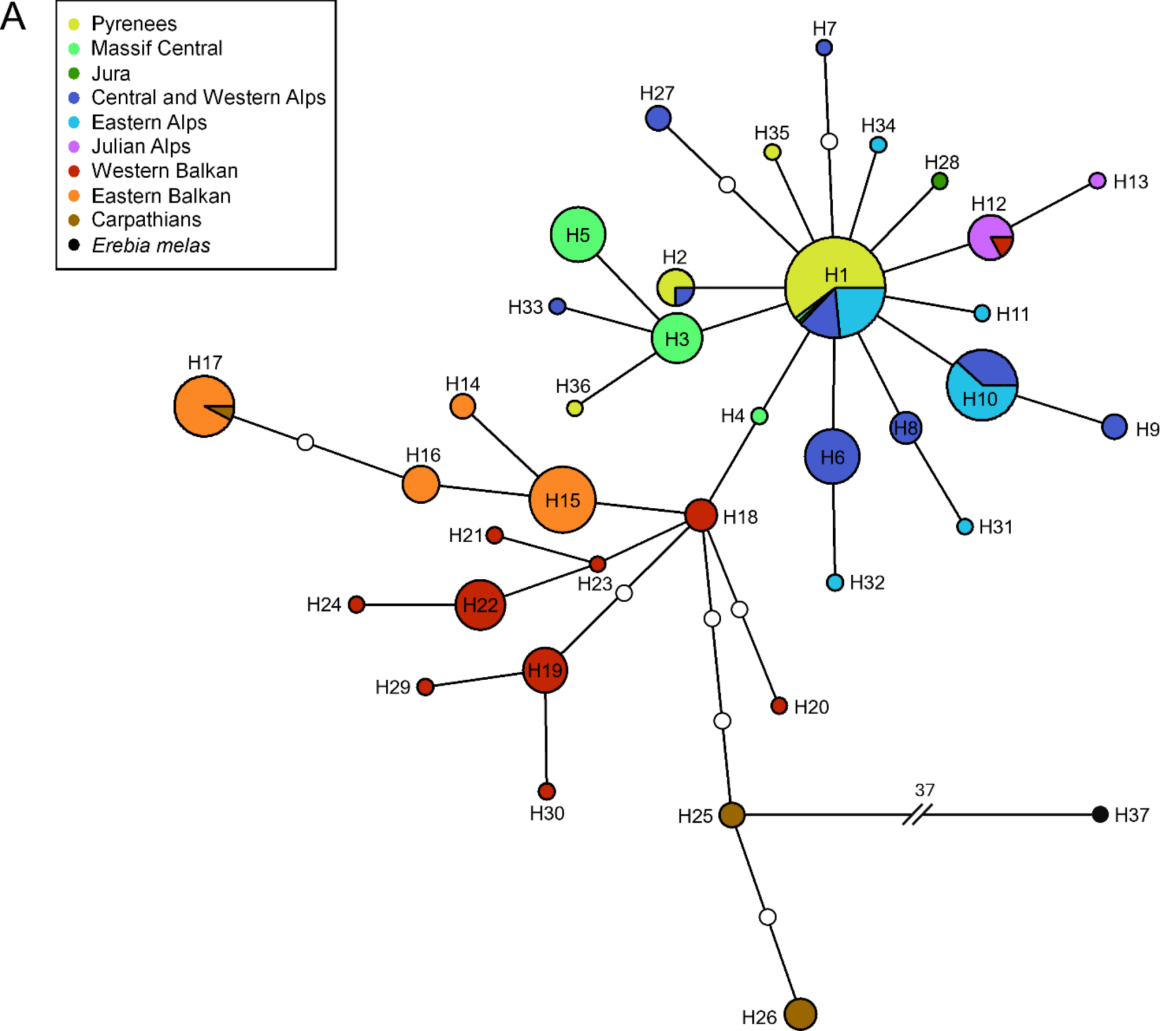
TCS haplotype network based on the barcode sequence (COI, 658 bp) of *Erebia oeme*. The small white circles on the links represent missing haplotypes. The corresponding colour codes to each region are given in the legend.

If only the barcode sequences (including the additional GenBank sequences) are considered (Fig. 4, S4), the Balkan group also exhibited a branched structure with no dominant haplotype. This group also included the haplotypes from the Carpathians: the haplotype in the Serbian Carpathians (i.e. south of Danube) was the eastern Balkan haplotype H17, the two haplotypes H25 and H26 from the southern Carpathians (Romania) built a subgroup distinguished by three mutational steps from the nearest western Balkan haplotype H18. The non-Balkan group also showed a star-like structure for the barcode fragment, with a single dominant haplotype (H1) including samples from the Pyrenees, Massif Central, Eastern Alps, and additional samples from the Jura and Western Alps. In contrast to the haplotype network based on COI and Cytb, the Julian Alps, together with a Slovenian sample from the western Balkans, were identified as derived haplotypes within the non-Balkan group and not as the link between non-Balkan and Balkan group.

**Fig. 5:**
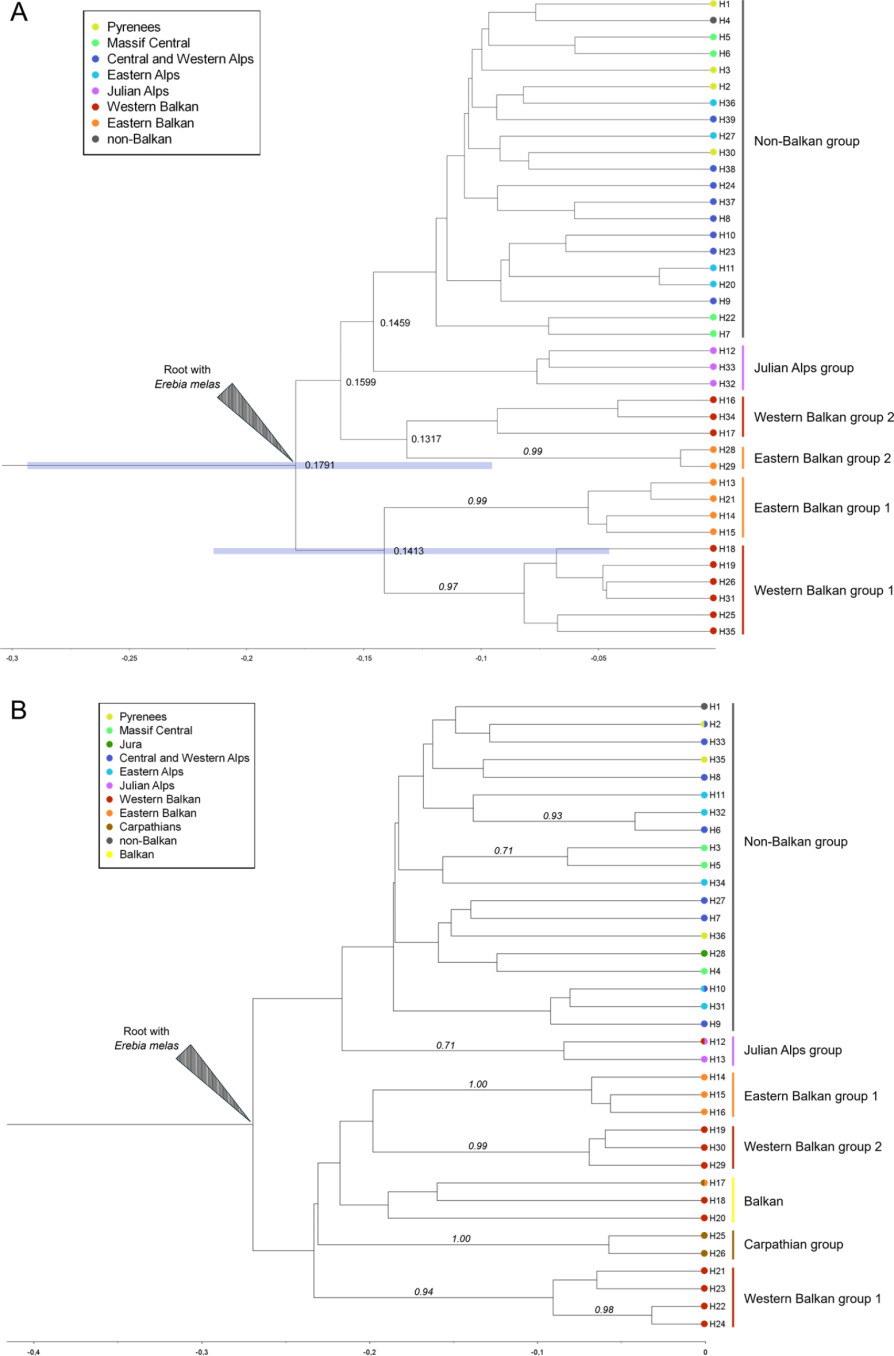
Bayesian phylogeny of *Erebia oeme*. (**A**) Based on mitochondrial haplotypes (COI, Cytb). (**B**) Based on the barcode haplotypes (COI, 568 bp) including sequences taken from GenBank. Numbers next to the nodes present their ages in million years with additional 95% highest posterior density for some node ages. Numbers on the branches present posterior probability numbers > 70%. The haplotypes are colour-coded according to the geographic affiliation of the included samples to a mountain region. The corresponding colour codes for each region are given in the legend.

Using all models recommended by AIC for the genes, the Bayesian analysis of the main dataset as well as the barcode dataset reached convergence after approximately 3,000,000 generations, with an ESS value of 3418 and 3497, respectively, for the posterior as well as 6067 and 8140 for the prior. The Bayesian phylogeny of the haplotypes of the main dataset revealed two groups that diverged approximately 180 ky ago (Fig. 5A). In contrast to the previous analyses, the first group included only samples from Vihren and Granchar from the eastern Balkan Peninsula (eastern Balkan group 1) as well as samples from Pošćenski Kraj, Vjetrena Brda and one sample from Kom Vasojevićki from the western Balkan Peninsula (Western Balkan group 1). All other samples were classified into the second group, which in turn had several subgroups. The first splitting subgroup within this second group contained specimens from the eastern and western Balkan Peninsula (i.e. eastern Balkan group 2, western Balkan group 2) and was sister to all samples from the Alps, including the Julian Alps, the Massif Central and the Pyrenees. If only these samples were considered, the Slovenian haplotypes (Julian Alps group) represented the sister group of the non-Balkan individuals (i.e., the non-Balkan group). Thus, the non-Balkan group, including and excluding the individuals from the Julian Alps, represented a monophyletic group, while the Balkan groups were paraphyletic. When considering the individuals from the respective mountain regions, all of them, except for the Julian Alps, were polyphyletic.

The Bayesian phylogeny of the haplotypes of the barcode dataset also revealed two groups. In contrast, here the first group included all haplotypes from the eastern and western Balkans and additionally the southern Carpathian haplotypes and can be classified into five subgroups (eastern Balkan group 1, western Balkan groups 1 and 2, Balkan group including eastern and western Balkan haplotypes, as well as southern Carpathian group). The second group contained all non-Balkan haplotypes (non-Balkan group) as well as the haplotypes of the Julian Alps including also one western Balkan sample from Slovenia (Julian Alps group). Similar to the phylogeny based on the main dataset, the individuals from the respective mountain regions were polyphyletic, except for the specimens from the Julian Alps, which were paraphyletic.

S-DIVA analysis generated with RASP was not able to detect an area of origin for *E. oeme* (**Figure S5**). Mismatch distribution analysis displayed a bimodal distribution (**Figure S6**). Except for the specimens from the eastern Balkan Peninsula, which offered a significant positive Tajima’s D value of 2.16, no further significant Tajima’s D values were detected (**Table S6**).

### *Wolbachia* infections, strain diversity and phylogeny

In total, 135 out of 166 *E. oeme* specimens (~ 81.3%) were infected with the parasitic endobacterium *Wolbachia* (**Table S7**), and three different *Wolbachia* strains were detected. The majority of *E. oeme* was infected with *Wolbachia* strain 1. Three *E. oeme* individuals from Lac Pavin (Massif Central) were infected with *Wolbachia* strain 2. *Wolbachia* strain 3 was detected in one specimen from Col de Pause (Pyrenees). These three *Wolbachia* strains showed pairwise genetic distances ranging from 0.080 between strains 1 and 3 to 0.204 between strains 2 and 3 (Table 4).

**Table 4:** Pairwise genetic distance of the four detected *Wolbachia* strains in *Erebia oeme.*

Wolbachia strain (host sample)	Strain 1	Strain 2	Strain 3
Strain 1 (EO001)	–		
Strain 2 (EO008)	0.156	–	
Strain 3 (EO126)	0.080	0.204	–

The Maximum likelihood phylogeny of all detected *Wolbachia* strains showed the closest genetic relationship between strain 1 and strain 3 and formed a sister group to strain 2 (**Figure S7**).

## Discussion

### Phylogeography of *Erebia oeme*

#### Genetic diversity and differentiation

The genetic diversity of the Balkan specimens was comparatively higher than that of the non-Balkan or Slovenian group (Table 3). For example, the nucleotide diversity of the Balkan group was about three to four times higher than the nucleotide diversity of the non-Balkan or Slovenian group. Haplotype number and diversity as well as segregating sites were also relatively high in the Balkan samples, even with considerably less samples included for the Balkan than for the non-Balkan group. Slovenian specimens showed lower values for diversity parameters compared to the Balkan and non-Balkan group, but this might be simply due to the limited number of samples analysed, as diversity parameters are expected to increase notably with increasing sample size.

The intraspecific diversity for the mitochondrial markers was about 1% (**Figure ****S1**), which is equivalent to a medium level of intraspecific genetic diversity in Lepidoptera^53^. Previous studies have shown that both lower and higher levels of intraspecific divergence occur in alpine butterfly species^7,9,54^. The maximum pairwise genetic distance was detected within the Balkan group between samples from Durmitor and Ruen, which indicates a subdivision of the Balkan group into different lineages. This hypothesis is supported by our other analyses, which also assumed more than one lineage within the Balkan Peninsula. In total, taking all analyses into account, two major Balkan lineages of *E. oeme* were distinguished. This goes in line with other studies that also revealed several genetic lineages at the Balkan Peninsula in other species^55–57^. In this context, eastern and western Balkan Peninsula often represent distinct genetic lineages due to the Central Balkan Depression^4^. In our data, two genetic lineages were detected in the Balkan Peninsula, but between different specimens from the eastern and western region (Fig. 5A). Thus, the first Balkan Peninsula lineage (i.e., western and eastern group 1) included specimens from Vihren, Granchar (both east) as well as Durmitor and one sample from Kom Vasojevićki (both west); the second Balkan Peninsula lineage (i.e., western and eastern group 2) contained samples from Ruen (east) and Kom Vasojevićki (west). The genetic differentiation within these two lineages was about 0.75% (lineage 1) and 0.6% (lineage 2). In the case of barcode sequences, however, these two lineages cannot be identified. This discrepancy can be attributed to the lower genetic resolution of the barcode phylogeny (658 bp) in comparison to our more comprehensive mitochondrial dataset (1809 bp). Therefore, the mitochondrial phylogeny is given greater weight.

In contrast, the non-Balkan and Julian Alps group both represented a separate lineage each. Although the non-Balkan group included samples from the Alps, Massif Central, Jura and Pyrenees, which often possess distinct lineages in alpine species^6,7,54^, no pronounced phylogeographic structure was identified for *E. oeme* all over these mountain ranges. Some geographically close populations, particularly in the western Alps and Massif Central, showed a slight clustering (Figs. 3 and 5), but the genetic differentiation among them is not sufficiently strong to justify discrete lineages (**Figure ****S1**). In the Julian Alps group, genetic differentiation might be limited by the small sample size.

The existence of potential subspecies could neither be proven nor denied due to a lack of morphological information about the specimens. The subspecies suggested in Sonderegger^58^ has no overlap with our genetic patterns, in particular as the mentioned subspecies are assumed to have partial sympatry. This might be explained by quick adaptation to local conditions so that in Pyrenees, Massif Central, Jura and Alps (except for the Julian Alps) no distinct morphological subspecies should be considered. However, intraspecific genetic differentiation in the Balkan region and Julian Alps is sufficiently pronounced so that a distinction at subspecies level may be justfied^7,59^. Nevertheless, additional research is required to provide further evidence.

#### Phylogeny and biogeography

The comparatively high level of genetic diversity and differentiation of the Balkan Peninsula group indicates a longer evolutionary history of Balkan individuals compared to those from Alps, Massif Central and Pyrenees, and consequently an origin of *E. oeme* located in the Balkan region. The Bayesian tree supports this hypothesis, revealing the two Balkan lineages as the most basal groups compared to the remaining samples (Fig. 5). As the internal structure at the western Balkan Peninsula is more pronounced than in its eastern parts, an area of origin in the western Balkan Peninsula is more likely. This origin contrasts with other mountain species with a similar distribution, most of which supposedly originated in the Alps^7,9,60^.

**Fig. 6:**
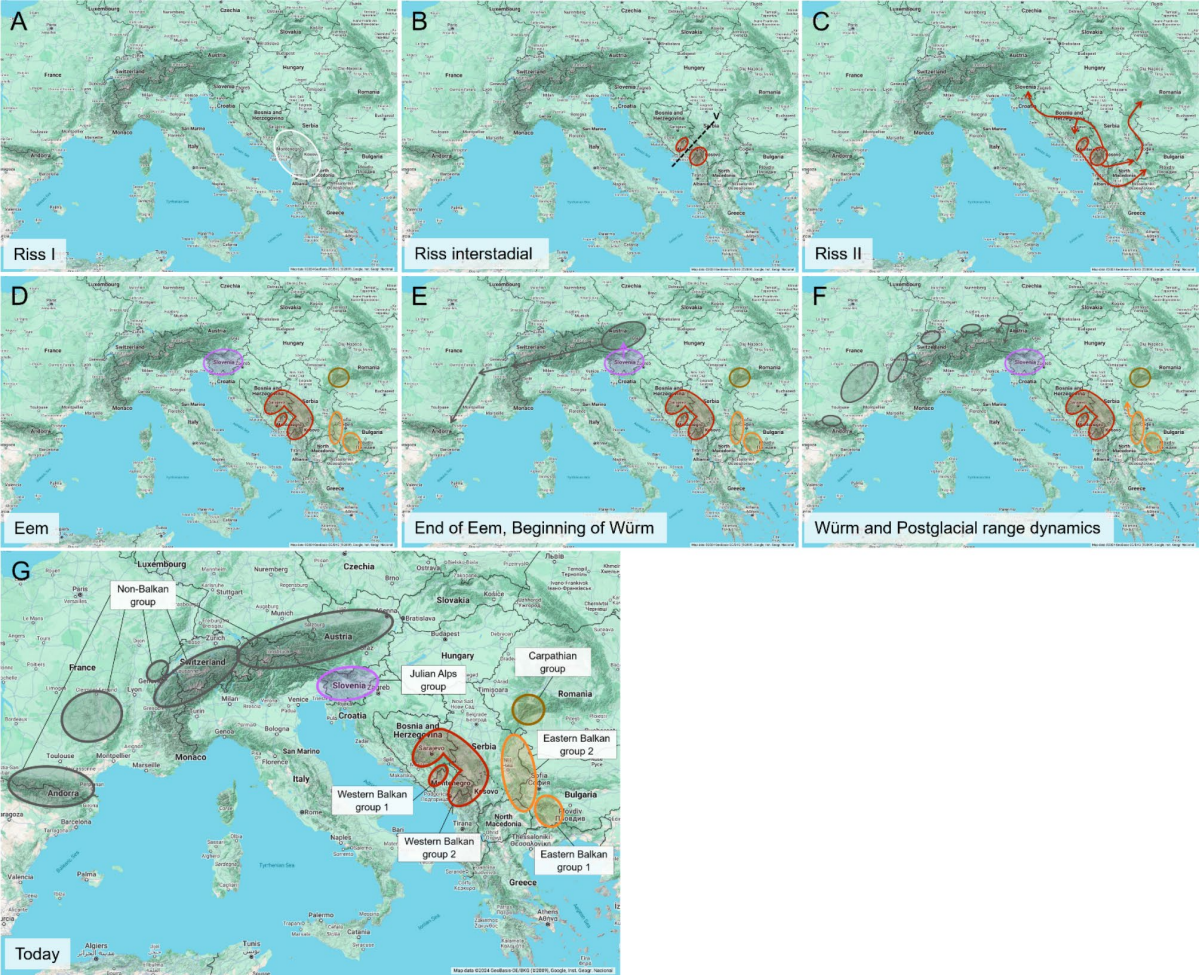
Biogeography of *Erebia oeme*. Genetic groups are colour coded based on their corresponding mountain range. White—group of origin, Dark grey—non-Balkan group, Lilac—Julian Alps group, Red—Western Balkan groups, Orange—Eastern Balkan groups, Brown—Carpathian group. Map created using Google My Maps (https://www.google.com/mymaps/) and further modified in PowerPoint (Microsoft PowerPoint for Microsoft 365 MSO, Version 2410 Build 16.0.18129.20158, 64-bit). Map data sources include: GeoBasis-DE/BKG 2024, Google, Instituto Geográfico Nacional.

The frequently assumed distribution fluctuations of other cold-adapted, alpine species can also be supported for *E. oeme*: During Riss I, a continuous population may have existed at the western Balkan Peninsula (Fig. 6A). This population apparently split into at least two lineages within the Dinaric Alps during an intensive interstadial of Riss glacial about 180 ky ago (Fig. 6B). This vicariance might have been triggered by warmer temperatures causing *E. oeme* to relocate to higher altitudes. It should be noted that, although the oldest recorded intraspecific split dates to 180 ky ago, the species itself most likely is much older, as supported by the estimated divergence time between *E. oeme* and the closely related *Erebia claudina* about 2 My ago^61^, i.e. about ten times older than the intraspecific differentiation. A likely reason for the much younger dating of the intraspecific differentiation possibly was a strong bottleneck in the Mindel-Riss interglacial, which might have strongly narrowed the genetic information and differentiation maybe due to strong reduction in its distribution and/or potential acquisition of *Wolbachia*.

During Riss II, the two emerged lineages most likely both spread to the eastern Balkan Peninsula; Balkan lineage 2 in addition also to Slovenia and to the southern Carpathians (Fig. 6C). Such biogeographic links between western Balkan Peninsula and south-eastern Alps as well as between the eastern Balkan Peninsula and the southern Carpathians have already been observed in different taxa, including several *Erebia* species^4,62^. In the Eem interglacial, two distinct lineages both must have existed at the western and eastern Balkan Peninsula, but in different mountain ranges, one of them in addition also in the Julian Alps and the southern Carpathians (Fig. 6D). During the transition from Eem interglacial to Würm glacial, between 120 and 70 ky ago, a dominant haplotype must have evolved in the eastern Alps, descending from immigrants out of Slovenia. This haplotype then must have rapidly spread throughout the Alps, as far as to the Massif Central and the Pyrenees (Fig. 6E). In the wake of this expansion process, the Pyrenees could have been colonised either via the Massif Central or directly from the western Alps.

The results of the mismatched distribution and Tajima’s D also supported these two range expansions (**Figure S6**,** Table S7**): the initial range expansion (right peak) of the mismatch distribution was associated with the expansion from the Western Balkan to the Eastern Balkan, the Carpathians and Slovenia. The second (left peak), more recent range expansion was related to the expansion of the non-Balkan group. This group also showed a strong negative Tajima’s D value. Although the observed value was not statistically significant, the results nonetheless supported the hypothesis of a range expansion of the non-Balkan group. In addition, the direction of the biogeographical pathway of *E. oeme* from the western Balkan Peninsula to the Alps, Massif Central and Pyrenees via Slovenia can be derived well from the NMDS and the PCA (Fig. 2, **Figure S2**): the two Slovenian samples are located mid-distance from the western Balkan Peninsula and the non-Balkan samples. Hence, the genetic proximity of Slovenian populations to either the Balkan or non-Balkan group is related to their geographic proximity to the Balkan Peninsula and Alps, respectively. For instance, Sija is geographically closer to the Balkan Peninsula than Mangart and also showed closer genetic cohesiveness to the Balkan group compared to Mangart.

During the Würm glaciation, *E. oeme* most likely survived in multiple glacial refugia around the northern and north-western Alps, Massif Central, north of the Pyrenees as well as in the mountains of the western and eastern Balkan Peninsula and in the southern Carpathians (Fig. 6F). These assumed refugia are partly in line with the centres of glacial survival of other widespread alpine species and are the basis for their today’s disjunct distribution in the western Palearctic mountain ranges (Fig. 6G)^3^. Southern Alpine refugia, which are often common for widespread alpine species^3^, are little likely in *E. oeme* referring to our data. The non-Balkan group showed a clear range expansion from glacial refugia across formerly glaciated mountain ranges, well reflected in the star-like structure of haplotypes in the network, even if not significantly supported by Tajima’s D values (**Table S6**). In contrast, the eastern Balkan Peninsula populations showed a significant, positive value of Tajima’s D, indicating range regression^4^. Despite this assumed range regression, the eastern Balkan Peninsula presumably is the origin of the secondary colonisation of the Serbian parts of the Carpathians due to the shared haplotype (Fig. 4) and the frequently observed biogeographical connection between these two mountain ranges^4^.

Since the last glacial period, individuals from the non-Balkan lineage have undergone separate evolutionary histories, which, however, were not long enough to result in pronounced genetic differentiation. During this period, a new *Wolbachia* strain was acquired in the Massif Central and, more recently, also in the Pyrenees, which in the long term may lead to stronger differentiation of the affected populations. Despite the disjunct distribution of *E. oeme*, secondary contact and intermixture was observed between populations in geographic proximity. For instance, the individual EO149 sampled in Kom Vasojevićki is genetically assigned to Balkan lineage 1, unlike the other specimens there, which belong to Balkan lineage 2.

### Prevalence, strain diversity and phylogeny of *Wolbachia* in *Erebia oeme*

Overall, more than 80% of the analysed *E. oeme* individuals were infected with *Wolbachia*. This prevalence is high compared to other infested taxa^63,64^, even in the genus *Erebia*, which generally provides high *Wolbachia* prevalence^7,18^. High prevalence may indicate an ancient relationship and co-adaptation between the host and *Wolbachia* as well as specific reproductive manipulation strategies of *Wolbachia*^64–66^. In *E. oeme*, *Wolbachia* strain 1 was widely detected in all regions and populations examined (**Table S7**). This implies that *Wolbachia* strain 1 invaded *E. oeme* before the first expansion out of the western Balkan Peninsula to its eastern parts and to Slovenia during the Riss glaciation, remaining in the species throughout time and space. Accordingly, *Wolbachia* must have survived in association with *E. oeme* in all of its refugia over the last two glacial-interglacial cycles. Multiple, more recent acquisitions of strain 1 after the expansion are much less likely, as the strain showed minor to no internal variability.

In addition to strain 1, two other strains were detected in *E. oeme*: strain 2 was found in three individuals from Lac Pavin (Massif Central) and strain 3 was present in one individual from Col de Pause (Pyrenees). The genetic distances of these strains to strain 1 were about 8% (Strain 1 and 3) and 15% (Strain 1 and 2), which confirms a parallel infection with distinct *Wolbachia* strains within one population. Such parallel infections with different *Wolbachia* strains are common within species and have also been detected within single populations^7,67,68^. At the level of the mitochondrial DNA, individuals with strain 2 or 3 do not differ from uninfected or strain 1 infected individuals. In addition, the presence of *Wolbachia* seems to have no recent effect on the genetic diversity of the mitochondrial markers in *E. oeme* (Table 2). The existing gene flow and the lack of genetic differentiation suggest that strains 2 and 3 were acquired most recently and argue against hybridisation or introgression for acquisition as well as against cytoplasmic incompatibility as a reproductive strategy of *Wolbachia* in this case. The transmission of these strains to individuals of *E. oeme* could have occurred, for example, through close ecological contact such as parasitism or sharing the same food source^69–71^.

## Conclusion

This study investigated the phylogeography of the Bright-eyed ringlet *E. oeme*. The genetic analyses demonstrated that the presumed intraspecific differentiation could be observed at the mitochondrial level. In total, four distinct lineages were genetically observed: two Balkan Peninsula lineages, one Julian Alps lineage and one lineage included the remaining samples from Alps, Massif Central, Jura and Pyrenees. The southern Carpathians (only barcoding region) might belong to one of the Balkan lineages or even might represent a lineage of its own. The observed intraspecific differentiation is sufficiently strong for a distinction at the subspecies level. However, confirming the suspected subspecies is challenging due to the lack of morphological data and limited sample size from some mountain regions (e.g., southern Carpathians, north-western Balkan Peninsula or south-western Alps) and cannot be conclusively clarified in this study. For this purpose, subsequent studies have to focus on the status of subspecies using both morphological and genetic data. The analysis of populations from regions with limited sample size in this study could also be useful to evaluate and complete the phylogeographic patterns described here.

However, the observed genetic differentiation was not detected in the tested nuclear markers. This indicates that *E. oeme* is either a recently diverged taxon, which, however, contradicts the splitting of *E. oeme* and *E. claudina* 2 My ago^61^, or more likely that there has been a strong bottleneck in its evolutionary history, resulting in a loss of genetic information and intraspecific differentiation. Potential causes may be a strong range reduction during the glacial-interglacial cycles and/or acquisition of *Wolbachia*.

Due to a pronounced internal genetic diversity and differentiation, *E. oeme* was hypothesised to originate from the western Balkan Peninsula. From this area of origin, *E. oeme* first spread to the eastern parts of the peninsula, to the southern Carpathians as well as to Slovenia during Riss glaciation and from there rapidly across the Alps to Massif Central and Pyrenees during the cool period between Eem interglacial and the onset of Würm glaciation.

The presence of *Wolbachia* was confirmed in approximately 80% of the individuals of *E. oeme*. The prevalence is comparably high, which can imply an ancient relationship or co-evolution between *Wolbachia* and its host. A total of three distinct strains was identified in *E. oeme*. However, there was no detected difference at the mitochondrial levels between individuals infected with different strains or uninfected individuals, indicating that *Wolbachia* apparently had no recently detectable impact on the phylogeography of *E. oeme*. However, in the long run, the individuals/populations with different *Wolbachia* infections may become more distinct from each other or from uninfected individuals, potentially leading to a more pronounced intraspecific differentiation.

## Electronic supplementary material

Below is the link to the electronic supplementary material.

Supplementary Material 1

## Data Availability

The datasets used and/or analysed during the current study are available from the corresponding author on reasonable request. All data generated or analysed during this study are included in this published article [and its supplementary information files]. The sequences are available in GenBank (PQ341286‒PQ341565, PQ354239‒PQ354407 and PQ345551‒PQ345716).
