# Supplementary material for: Phylogeographic analyses reveal recent dispersal and multiple Wolbachia infections of the bright-eyed ringlet Erebia oeme within the European mountain systems
Source: Sci Rep. 2025 Jan 14;15:1956. doi: 10.1038/s41598-024-84551-5 (PMC11733208; doi:10.1038/s41598-024-84551-5)
Supplement: Supplementary file 1 — Supplementary Material 1 [file 41598_2024_84551_MOESM1_ESM.docx]

Phylogeographic analyses reveal recent dispersal and multiple *Wolbachia* infections of the Bright-eyed ringlet *Erebia oeme* within the European mountain systems

**Authors**: Valentine Mewis^1^*, Martin Wendt^2^, Thomas Schmitt^1,3^**

^1^Senckenberg Deutsches Entomologisches Institut, Systematik und Biogeographie, Eberswalder Str. 90, 15374 Müncheberg, Germany. ^2^Leibniz-Zentrum für Agrarlandschaftsforschung (ZALF) e.V., Eberswalder Str. 84, 15374 Müncheberg, Germany. ^3^Entomology and Biogeography, Institute of Biochemistry and Biology, Faculty of Science, University of Potsdam, 14476 Potsdam, Germany. Emails: *valentine.mewis@senckenberg.de, **thomas.schmitt@senckenberg.de

# Supplementary

**Table S1**: List of the populations of *Erebia oeme* with number of individuals and ID number of the included individuals for each population as well as the geographical information. AUT – Austria, BGR – Bulgaria, CHE – Switzerland, ESP – Spain, FRA – France, MNE – Montenegro and SVN – Slovenia.

| Population | Number of individuals | ID of the individuals | State | Region | Location | Collection date |
| --- | --- | --- | --- | --- | --- | --- |
| EO-ESTO | 10 | EO001-2, EO025-32 | ESP | Pyrenees | Toses | 13.07.2013 |
| EO-FRBM | 10 | EO131-140 | FRA | Pyrenees | Las Planes near Bourg-Madame | 11.07.2013 |
| EO-FRCP | 10 | EO121-130 | FRA | Pyrenees | Col de Pause | 18.07.2013 |
| EO-FRCT | 10 | EO003-4, EO089-96 | FRA | Pyrenees | Cauterets | 16.07.2013 |
| EO-FRCS | 10 | EO005-6, EO049-56 | FRA | Massif Central | Col des Supeyres | 10.07.2013 |
| EO-FRLP | 10 | EO007-8, EO081-88 | FRA | Massif Central | Lac Pavin | 13.08.2008 |
| EO-FRCA | 10 | EO009-10, EO073-80 | FRA | Central and Western Alps | Col des Aravis | 28.07.2005 |
| EO-CHVI | 1 | EO156 | CHE | Central and Western Alps | Val-d'Illiez | 08.08.2005 |
| EO-CHLU | 12 | EO157-168 | CHE | Central and Western Alps | Lütschental | 22.07.2013 |
| EO-CHPA | 10 | EO011-12, EO057-64 | CHE | Central and Western Alps | Partnun | 14.07.2005 |
| EO-ATWK | 10 | EO101-110 | AUT | Eastern Alps | Sonnenstein, Wilder Kaiser | 02.08.2006 |
| EO-ATKS | 1 | EO155 | AUT | Eastern Alps | Kolm-Saigurn | 20.07.2007 |
| EO-ATHA | 10 | EO013-14, EO033-40 | AUT | Eastern Alps | Halltal | 12.07.2007 |
| EO-SVMA | 2 | EO015-16 | SVN | Julian Alps | Mangart | 21.07.2011 |
| EO-SVSI | 2 | EO145-146 | SVN | Julain Alps | Sija | 22.07.2011 |
| EO-BGGR | 10 | EO017-18, EO065-72 | BGR | Eastern Balkan | Granchar | 31.07.2010 |
| EO-BGVI | 13 | EO019-20, EO041-44, EO047-48, EO152-154 | BGR | Eastern Balkan | Vihren | 02.08.2010 |
| EO-BGRU | 10 | EO111-120 | BGR | Eastern Balkan | Ruen | 03.08.2011 |
| EO-MNKV | 7 | EO021-22, EO147-151 | MNE | Western Balkan | Kom Vasojevićki | 29.07.2014 |
| EO-MNDU | 10 | EO023-24, EO097-100, EO141-144 | MNE | Western Balkan | Pošćenski Kraj, Vjetrena Brda | 02. & 03.08.2014 |
| Outgroup | 1 | *Erebia melas* | MNE | Western Balkan | Pošćenski Kraj | 02.08.2014 |

**Table S2**: List of the additional individuals of *Erebia oeme* with GenBank accession numbers, sequence length of the Barcode fragment, geographic information and reference, AND – Andorra, AUT – Austria, BIH – Bosnia and Herzegovina, BGR – Bulgaria, CHE – Switzerland, ESP – Spain, FRA – France, GER – Germany, ITA – Italia, NMK – North Macedonia, ROU – Romania, SRB - Serbia and SVN – Slovenia

| GenBank Accession number | Sequence length [bp] | State | Region | Location | Reference |
| --- | --- | --- | --- | --- | --- |
| FJ938192 | 627 | ROU | Southern Carpathians | Transylvania, Scorota Valley (Retezat Mts.) | [1] |
| FJ938193 | 658 | ROU | Southern Carpathians | Transylvania, Scorota Valley (Retezat Mts.) | [1] |
| FJ938194 | 658 | ROU | Southern Carpathians | Transylvania, Scorota Valley (Retezat Mts.) | [1] |
| FJ938195 | 658 | ROU | Southern Carpathians | Transylvania, Scorota Valley (Retezat Mts.) | [1] |
| FJ938196 | 658 | ROU | Southern Carpathians | Transylvania, Scorota Valley (Retezat Mts.) | [1] |
| MW501758 | 658 | SRB | Serbian Carpathians | Crni Vrh | [2] |
| MW502572 | 658 | SRB | Eastern Balkan | Babin Zub | [2] |
| MW501977 | 658 | BGR | Eastern Balkan | Sandanski, Orelek (Pirin Mts.) | [2] |
| MW499642 | 658 | BGR | Eastern Balkan | Berkovitsa, Road to Kom peak (Stara Planina) | [2] |
| MW503110 | 658 | BGR | Eastern Balkan | Yakoruda, Road to Belmeken (Rila Mts.) | [2] |
| MW501421 | 658 | NMK | Western Balkan | Mavrovo NP, Korab, Korabska jezero, Kobilino pole | [2] |
| MW502697 | 658 | NMK | Western Balkan | Mavrovo NP, Korab, Korabska jezero, Kobilino pole | [2] |
| MW501254 | 658 | NMK | Western Balkan | Gostivar, Sharr Mt., Rudoka | [2] |
| MW500552 | 614 | NMK | Western Balkan | Bistra Mt. (Mala Reka) | [2] |
| MW502613 | 658 | BIH | Western Balkan | Jablanica, Cvrsnica Mts. | [2] |
| MW501426 | 658 | BIH | Western Balkan | Cvrsnica | [2] |
| MW499522 | 658 | SVN | Western Balkan | Caven | [2] |
| MW499363 | 658 | ITA | Julian Alps | Udine, Jof di Montasio | [2] |
| MW502973 | 658 | ITA | Julian Alps | Udine, Jof di Montasio | [2] |
| MW499171 | 658 | AUT | Eastern Alps | Southern side of Dachstein (Gletschbahn Ramsau to Suedwandhuette) | [2] |
| MN142023 | 658 | AUT | Eastern Alps | Steiermark, Schwarze Walster | [3] |
| MN141247 | 658 | AUT | Eastern Alps | Oberoesterreich, Hopfing | [3] |
| MN143720 | 658 | AUT | Eastern Alps | Tirol, Nordtirol, Achselkopf | [3] |
| MN141172 | 631 | AUT | Eastern Alps | Tirol, Nordtirol, Achselkopf | [3] |
| MN140779 | 658 | AUT | Eastern Alps | Tirol, Nordtirol, Achselkopf | [3] |
| MN139582 | 658 | AUT | Eastern Alps | Niederoesterreich, Bodingbach, Handhab | [3] |
| MN139039 | 658 | AUT | Eastern Alps | Steiermark, Schwarze Walster | [3] |
| KP253354 | 658 | AUT | Eastern Alps | Vorarlberg, Hochgerach/ Schnifis | [4] |
| KP253190 | 658 | AUT | Eastern Alps | Vorarlberg, E Laguzalpe/ Marul | [4] |
| JF415703 | 658 | GER | Eastern Alps | Bavaria, Oberbayern, Karwendelgebirge, Mittenwald, Vereinsalm | [5] |
| JF415702 | 658 | GER | Eastern Alps | Bavaria, Oberbayern, Samerberg, Lederstuben | [5] |
| KX040697 | 658 | GER | Eastern Alps | Bavaria, Oberbayern, Berchtesgadener Land, Schoenau, Jenner/Stahlhaus | [6] |
| KX040421 | 658 | GER | Eastern Alps | Bavaria, Schwaben, Oberallgaeu, Oberstdorf, Oytal | [6] |
| JN278903 | 658 | GER | Eastern Alps | Bavaria, Unterwössen/Marquartstein | iBOL Data Release |
| MK186350 | 654 | CHE | Central and Western Alps | Graubunden, Piz Beverin | [7] |
| MK186349 | 654 | CHE | Central and Western Alps | Bern, Lauenen | [7] |
| MK186348 | 618 | CHE | Central and Western Alps | Valais, Derborence | [7] |
| MW503442 | 658 | CHE | Central and Western Alps | Hinterrhein, Splugenpass | [2] |
| MW502039 | 658 | CHE | Central and Western Alps | Interlaken, Bort | [2] |
| MN139287 | 658 | CHE | Central and Western Alps | Bern Canton, Adelboden | [3] |
| MW502003 | 658 | FRA | Jura | Septmoncel | [2] |
| MW503161 | 658 | FRA | Jura | Septmoncel | [2] |
| GU676018 | 658 | FRA | Pyrenees | Col de Puymorens | iBOL Data Release |
| KR138834 | 658 | FRA | Pyrenees | Languedoc, Ariege 09, Ustou | [8] |
| KR138752 | 658 | FRA | Pyrenees | Languedoc, Ariege 09, Ustou | [8] |
| GU676015 | 658 | AND | Pyrenees | Grau Roig | iBOL Data Release |
| KU707843 | 658 | ESP | Pyrenees | Catalonia, Pallars Sobir, Planes de Son | BOLD Student Data Portal Data Release |
| JN827884 | 658 | ESP | Pyrenees | Catalonia, Lleida, Bassa d`Arres, Vall d`Aran | [9] |
| JN827883 | 658 | ESP | Pyrenees | Catalonia, Lleida, Bassa d`Arres, Vall d`Aran | [9] |
| JN827882 | 658 | ESP | Pyrenees | Catalonia, Lleida, Bassa d`Arres, Vall d`Aran | [9] |
| JN827881 | 658 | ESP | Pyrenees | Catalonia, Lleida, Bassa d`Arres, Vall d`Aran | [9] |
| JN827880 | 658 | ESP | Pyrenees | Catalonia, Lleida, Bassa d`Arres, Vall d`Aran | [9] |
| HM901830 | 637 | ESP | Pyrenees | Catalonia, Lleida, Begos | iBOL Data Release |
| HM901829 | 658 | ESP | Pyrenees | Catalonia, Lleida, Begos | iBOL Data Release |
| HM901505 | 658 | ESP | Pyrenees | Catalonia, Lleida, Begos | iBOL Data Release |
| KP870780 | 658 | ESP | Pyrenees | Catalonia, Girona, Vallsabollera, La Cerdanya | [10] |

**Table S3**: Modification of the “DNA Extraction and Purification from Tissue” protocol for DNA extraction.

| Step | Modification |
| --- | --- |
| Step 3 | No vortexting, inverting instead |
| Step 5 and 6 | Was not executed |
| Step 7 and 9 | No vortexting, inverting instead |

**Table** **S4**: List of used primers and PCR protocols for the tested nuclear markers.

| Marker | Primer | | | | PCR protocol |
| --- | --- | --- | --- | --- | --- |
|  | **Name** | **Direction** | **Sequence** | **Reference** |  |
| CAD | 743nF-ino | forward | 5’-GGIGTIACIACIGCITGYTTYGARCC-3’ | [11,12] | 5' 95°C, 40x (30'' 95°C, 90" 55°C, 90" 72°C) 30' 68°C |
|  | 1028R-ino | reverse | 5’-TTRTTIGGIARYTGICCICCCAT-3’ |  |  |
| EF1α | M44-1 | forward | 5’-GCTGAGCGYGARCGTGGTATCAC-3’ | [13,14] | 5' 95°C, 40x (30'' 95°C, 90" 55°C, 90" 72°C) 30' 68°C or  5' 95°C, 40x (30'' 95°C, 90" 58-0.1°C/cycle, 60" 72°C) 30' 68°C |
|  | EFrcM4 | reverse | 5’-ACAGCVACKGTYTGYCTCATRTC-3’ | [13,15] |  |
| GAPDH | Frigga | forward | 5’-AARGCTGGRGCTGAATATGT-3’ | [15] | 5' 95°C, 40x (30'' 95°C, 90" 55°C, 90" 72°C) 30' 68°C |
|  | Burre | reverse | 5’-GWTTGAATGTACTTGATRAGRTC-3’ |  |  |
| IDH | deg27F-ino | forward | 5’-GGWGAYGARATGACIAGRATHATHTGG-3’ | [12] | 5' 95°C, 40x (30'' 95°C, 90" 50°C, 60" 72°C) 30' 68°C |
|  | degR-ino | reverse | 5’-TTYTTRCAIGCCCAIACRAAICCICC-3’ |  |  |
| MDH | MDH_F | forward | 5’-GAYATNGCNCCNATGATGGGNGT-3’ | [15] | 5' 95°C, 38x (30'' 95°C, 90" 53°C, 90" 72°C) 30' 68°C |
|  | MDH_R | reverse | 5’-AGNCCYTCNACDATYTTCCAYTT-3’ |  |  |
| NC | NC_F | forward | 5’-GATGAAGAAAAYCCHAARAARTTYTT-3’ | [16] | 5' 95°C, 40x (30'' 95°C, 90" 55°C, 90" 72°C) 30' 68°C |
|  | NC_R | reverse | 5’-ACWATDGACCARTGGAARTTCATDGC-3’ |  |  |
| RPS5 | RpS5F | forward | 5’-ATGGCNGARGARAAYTGGAAYGA-3’ | [15] | 5' 95°C, 40x (30'' 95°C, 90" 55°C, 90" 72°C) 30' 68°C |
|  | RpS5R | reverse | 5’-TTRGAYTTRGCAACACG-3’ |  |  |
| WG | LepWg1 | forward | 5’-GARTGYAARTGYCAYGGYATGTCTGG-3’ | [15,17] | 5' 95°C, 40x (30'' 95°C, 90" 50°C, 60" 72°C) 30' 68°C |
|  | LepWg2 | reverse | 5’-ACTICGCARCACCARTGGAATGTRCA-3’ |  |  |


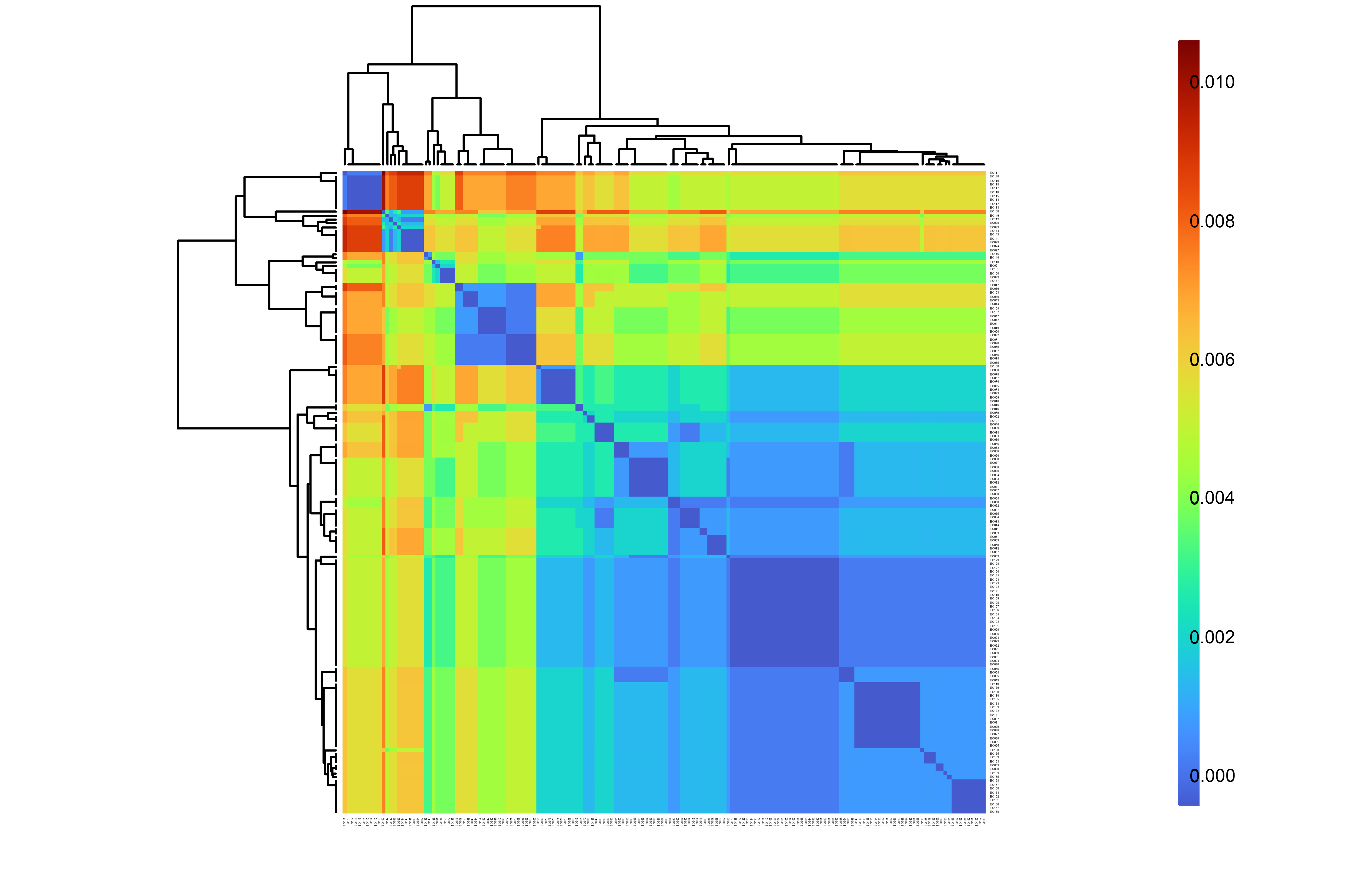


**Figure S1**: Heatmap presenting the pairwise genetic distances of *Erebia oeme* including two mitochondrial genes (COI and Cytb) with dendrogram. The colour codes of the genetic distances are shown in the legend.

**Figure S2**: Principal Component Analysis of *Erebia oeme* from the different mountain regions. The corresponding colour codes to each region are given in the legend.

MNDU

MNKV

BGRU

BGGR

BGVI

FRCS

FRLP

FRCT

FRCP

FRBM

FRCS

ESTO

ESTO

FRBM

ATKS

CHLU

ATWK

ATHA

SVSI

SVMA

FRCA

CHPA

CHVI

**Figure S3**: Principal Component Analysis of *Erebia oeme* from the different populations. The corresponding colour codes to each population are given in the legend.

**Table S5**: Haplotypes for each sample based on the mitochondrial markers COI and Cytb of *Erebia oeme* sorted by populations

| Sample | Haplotype | Sample | Haplotype | Sample | Haplotype |
| --- | --- | --- | --- | --- | --- |
| EO001 | I (H1) | EO085 | VII (H7) | EO039 | XX (H20) |
| EO002 | II (H2) | EO086 | VII (H7) | EO040 | XX (H20) |
| EO025 | I (H1) | EO087 | VII (H7) | EO015 | XII (H12) |
| EO026 | I (H1) | EO088 | VII (H7) | EO016 | XII (H12) |
| EO027 | I (H1) | EO009 | VIII (H8) | EO145 | XXXII (H32) |
| EO028 | I (H1) | EO010 | VIII (H8) | EO146 | XXXIII (H33) |
| EO029 | I (H1) | EO073 | VIII (H8) | EO017 | XIII (H13) |
| EO030 | IV (H4) | EO074 | VIII (H8) | EO018 | XIV (H14) |
| EO031 | I (H1) | EO075 | VIII (H8) | EO065 | XIV (H14) |
| EO032 | I (H1) | EO076 | VIII (H8) | EO066 | XIV (H14) |
| EO131 | I (H1) | EO077 | VIII (H8) | EO067 | XIV (H14) |
| EO132 | I (H1) | EO078 | VIII (H8) | EO068 | XIV (H14) |
| EO133 | I (H1) | EO079 | XXIV (H24) | EO069 | XIII (H13) |
| EO134 | I (H1) | EO080 | VIII (H8) | EO070 | XIV (H14) |
| EO135 | I (H1) | EO156 | XXXVII (H37) | EO071 | XIV (H14) |
| EO136 | I (H1) | EO157 | XXXVIII (H38) | EO072 | XIV (H14) |
| EO137 | II (H2) | EO158 | XXXVIII (H38) | EO019 | XV (H15) |
| EO138 | I (H1) | EO159 | XXXIX (H39) | EO020 | XV (H15) |
| EO139 | I (H1) | EO160 | XXXVIII (H38) | EO041 | XV (H15) |
| EO140 | I (H1) | EO161 | XXXVIII (H38) | EO042 | XV (H15) |
| EO121 | IV (H4) | EO162 | XXXVIII (H38) | EO043 | XXI (H21) |
| EO122 | IV (H4) | EO163 | XXXIX (H39) | EO044 | XXI (H21) |
| EO123 | IV (H4) | EO164 | XXXVIII (H38) | EO047 | XV (H15) |
| EO124 | IV (H4) | EO165 | XXXIX (H39) | EO048 | XXI (H21) |
| EO125 | IV (H4) | EO166 | XXXVIII (H38) | EO152 | XV (H15) |
| EO126 | IV (H4) | EO167 | XXXVIII (H38) | EO153 | XXI (H21) |
| EO127 | IV (H4) | EO168 | XXXVIII (H38) | EO154 | XV (H15) |
| EO128 | IV (H4) | EO011 | IX (H9) | EO111 | XXVIII (H28) |
| EO129 | IV (H4) | EO012 | X (H10) | EO112 | XXIX (H29) |
| EO130 | XXX (H30) | EO057 | X (H10) | EO113 | XXIX (H29) |
| EO003 | III (H3) | EO058 | X (H10) | EO114 | XXIX (H29) |
| EO004 | IV (H4) | EO059 | X (H10) | EO115 | XXIX (H29) |
| EO089 | IV (H4) | EO060 | XXIII (H23) | EO116 | XXIX (H29) |
| EO090 | III (H3) | EO061 | X (H10) | EO117 | XXIX (H29) |
| EO091 | IV (H4) | EO062 | XXIII (H23) | EO118 | XXIX (H29) |
| EO092 | IV (H4) | EO063 | IX (H9) | EO119 | XXIX (H29) |
| EO093 | IV (H4) | EO064 | XXIII (H23) | EO120 | XXIX (H29) |
| EO094 | IV (H4) | EO101 | IV (H4) | EO021 | XVI (H16) |
| EO095 | IV (H4) | EO102 | IV (H4) | EO022 | XVII (H17) |
| EO096 | IV (H4) | EO103 | XXVII (H27) | EO147 | XVII (H17) |
| EO005 | V (H5) | EO104 | IV (H4) | EO148 | XXXIV (H34) |
| EO006 | VI (H6) | EO105 | IV (H4) | EO149 | XXXV (H35) |
| EO049 | V (H5) | EO106 | IV (H4) | EO150 | XVII (H17) |
| EO050 | VI (H6) | EO107 | IV (H4) | EO151 | XVII (H17) |
| EO051 | IV (H4) | EO108 | IV (H4) | EO023 | XVIII (H18) |
| EO052 | VI (H6) | EO109 | IV (H4) | EO024 | XIX (H19) |
| EO053 | XXII (H22) | EO110 | IV (H4) | EO097 | XIX (H19) |
| EO054 | V (H5) | EO155 | XXXVI (H36) | EO098 | XXV (H25) |
| EO055 | VI (H6) | EO013 | XI (H11) | EO099 | XIX (H19) |
| EO056 | V (H5) | EO014 | XI (H11) | EO100 | XXVI (H26) |
| EO007 | VII (H7) | EO033 | XX (H20) | EO141 | XIX (H19) |
| EO008 | VII (H7) | EO034 | XI (H11) | EO142 | XXXI (H31) |
| EO081 | VII (H7) | EO035 | XI (H11) | EO143 | XIX (H19) |
| EO082 | VII (H7) | EO036 | XX (H20) | EO144 | XIX (H19) |
| EO083 | VII (H7) | EO037 | XI (H11) |  |  |
| EO084 | VII (H7) | EO038 | XX (H20) |  |  |

**Table S6**: Haplotypes for each sample based on the barcode sequence of *Erebia oeme*

| Sample | Haplotype | Sample | Haplotype | Sample | Haplotype |
| --- | --- | --- | --- | --- | --- |
| EO001 | I (H1) | EO160 | I (H1) | EO120 | XVII (H17) |
| EO002 | II (H2) | EO161 | I (H1) | EO021 | XVIII (H18) |
| EO025 | I (H1) | EO162 | I (H1) | EO022 | XIX (H19) |
| EO026 | I (H1) | EO163 | VIII (H8) | EO147 | XIX (H19) |
| EO027 | I (H1) | EO164 | I (H1) | EO148 | XX (H20) |
| EO028 | I (H1) | EO165 | VIII (H8) | EO149 | XXI (H21) |
| EO029 | I (H1) | EO166 | I (H1) | EO150 | XIX (H19) |
| EO030 | IV (H4) | EO167 | I (H1) | EO151 | XIX (H19) |
| EO031 | I (H1) | EO168 | I (H1) | EO023 | XXII (H22) |
| EO032 | I (H1) | EO011 | IX (H9) | EO024 | XXII (H22) |
| EO131 | I (H1) | EO012 | X (H10) | EO097 | XXII (H22) |
| EO132 | I (H1) | EO057 | X (H10) | EO098 | XXIII (H23) |
| EO133 | I (H1) | EO058 | X (H10) | EO099 | XXII (H22) |
| EO134 | I (H1) | EO059 | X (H10) | EO100 | XXIV (H24) |
| EO135 | I (H1) | EO060 | X (H10) | EO141 | XXII (H22) |
| EO136 | I (H1) | EO061 | X (H10) | EO142 | XXII (H22) |
| EO137 | II (H2) | EO062 | X (H10) | EO143 | XXII (H22) |
| EO138 | I (H1) | EO063 | IX (H9) | EO144 | XXII (H22) |
| EO139 | I (H1) | EO064 | X (H10) | FJ938192 | XXV (H25) |
| EO140 | I (H1) | EO101 | I (H1) | FJ938193 | XXV (H25) |
| EO121 | I (H1) | EO102 | I (H1) | FJ938194 | XXVI (H26) |
| EO122 | I (H1) | EO103 | XI (H11) | FJ938195 | XXVI (H26) |
| EO123 | I (H1) | EO104 | I (H1) | FJ938196 | XXVI (H26) |
| EO124 | I (H1) | EO105 | I (H1) | MW501758 | XVII (H17) |
| EO125 | I (H1) | EO106 | I (H1) | MW502572 | XVII (H17) |
| EO126 | I (H1) | EO107 | I (H1) | MW501977 | XV (H15) |
| EO127 | I (H1) | EO108 | I (H1) | MW499642 | XVII (H17) |
| EO128 | I (H1) | EO109 | I (H1) | MW503110 | XV (H15) |
| EO129 | I (H1) | EO110 | I (H1) | MW501421 | XXIX (H29) |
| EO130 | I (H1) | EO155 | I (H1) | MW502697 | XIX (H19) |
| EO003 | I (H1) | EO013 | X (H10) | MW501254 | XIX (H19) |
| EO004 | I (H1) | EO014 | X (H10) | MW500552 | XXX (H30) |
| EO089 | I (H1) | EO033 | X (H10) | MW502613 | XVIII (H18) |
| EO090 | I (H1) | EO034 | X (H10) | MW501426 | XVIII (H18) |
| EO091 | I (H1) | EO035 | X (H10) | MW499522 | XII (H12) |
| EO092 | I (H1) | EO036 | X (H10) | MW499363 | XII (H12) |
| EO093 | I (H1) | EO037 | X (H10) | MW502973 | XII (H12) |
| EO094 | I (H1) | EO038 | X (H10) | MW499171 | I (H1) |
| EO095 | I (H1) | EO039 | X (H10) | MN142023 | I (H1) |
| EO096 | I (H1) | EO040 | X (H10) | MN141247 | XXXII (H32) |
| EO005 | III (H3) | EO015 | XII (H12) | MN143720 | XXXI (H31) |
| EO006 | III (H3) | EO016 | XII (H12) | MN141172 | X (H10) |
| EO049 | III (H3) | EO145 | XIII (H13) | MN140779 | X (H10) |
| EO050 | III (H3) | EO146 | XII (H12) | MN139582 | I (H1) |
| EO051 | I (H1) | EO017 | XIV (H14) | MN139039 | I (H1) |
| EO052 | III (H3) | EO018 | XV (H15) | KP253354 | I (H1) |
| EO053 | IV (H4) | EO065 | XV (H15) | KP253190 | I (H1) |
| EO054 | III (H3) | EO066 | XV (H15) | JF415703 | X (H10) |
| EO055 | III (H3) | EO067 | XV (H15) | JF415702 | I (H1) |
| EO056 | III (H3) | EO068 | XV (H15) | KX040697 | XXXIV (H34) |
| EO007 | V (H5) | EO069 | XIV (H14) | KX040421 | I (H1) |
| EO008 | V (H5) | EO070 | XV (H15) | JN278903 | I (H1) |
| EO081 | V (H5) | EO071 | XV (H15) | MK186350 | XXVII (H27) |
| EO082 | V (H5) | EO072 | XV (H15) | MK186349 | II (H2) |
| EO083 | V (H5) | EO019 | XV (H15) | MK186348 | XXXIII (H33) |
| EO084 | V (H5) | EO020 | XV (H15) | MW503442 | XXVII (H27) |
| EO085 | V (H5) | EO041 | XV (H15) | MW502039 | I (H1) |
| EO086 | V (H5) | EO042 | XV (H15) | MN139287 | I (H1) |
| EO087 | V (H5) | EO043 | XVI (H16) | MW502003 | XXVIII (H28) |
| EO088 | V (H5) | EO044 | XVI (H16) | MW503161 | I (H1) |
| EO009 | VI (H6) | EO047 | XV (H15) | GU676018 | I (H1) |
| EO010 | VI (H6) | EO048 | XVI (H16) | KR138834 | I (H1) |
| EO073 | VI (H6) | EO152 | XV (H15) | KR138752 | XXXV (H35) |
| EO074 | VI (H6) | EO153 | XVI (H16) | GU676015 | I (H1) |
| EO075 | VI (H6) | EO154 | XV (H15) | KU707843 | I (H1) |
| EO076 | VI (H6) | EO111 | XVII (H17) | JN827884 | I (H1) |
| EO077 | VI (H6) | EO112 | XVII (H17) | JN827883 | I (H1) |
| EO078 | VI (H6) | EO113 | XVII (H17) | JN827882 | I (H1) |
| EO079 | VII (H7) | EO114 | XVII (H17) | JN827881 | I (H1) |
| EO080 | VI (H6) | EO115 | XVII (H17) | JN827880 | I (H1) |
| EO156 | VI (H6) | EO116 | XVII (H17) | HM901830 | XXXVI (H36) |
| EO157 | I (H1) | EO117 | XVII (H17) | HM901829 | I (H1) |
| EO158 | I (H1) | EO118 | XVII (H17) | HM901505 | I (H1) |
| EO159 | VIII (H8) | EO119 | XVII (H17) | KP870780 | II (H2) |
| *Erebia melas* | XXXVII (H37) |  |  |  |  |


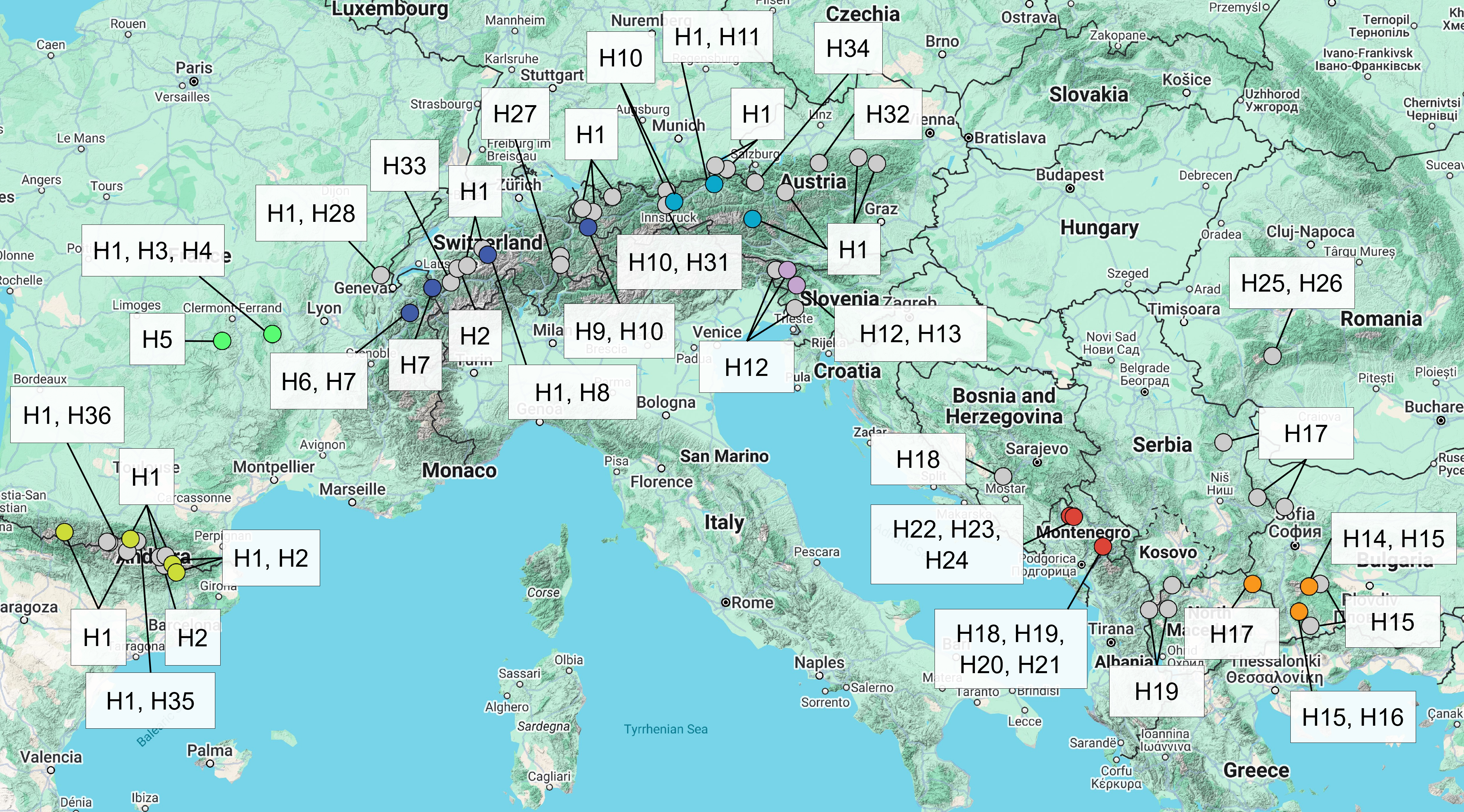


**Figure S4**: Geographic distribution of the haplotypes based on the barcode sequence of *Erebia oeme*. Chartreuse – Pyrenees, Green – Massif Central, Blue – Central and Western Alps, Light blue – Eastern Alps, Lilac – Julian Alps, Red – Western Balkan Peninsula, Orange – Eastern Balkan Peninsula, Grey – GenBank sequences. Map created using Google My Maps (<https://www.google.com/mymaps/>) and further modified in PowerPoint (Microsoft PowerPoint for Microsoft 365 MSO, Version 2410 Build 16.0.18129.20158, 64-bit). Map data sources include: GeoBasis-DE/BKG 2024, Google, Instituto Geográfico Nacional.




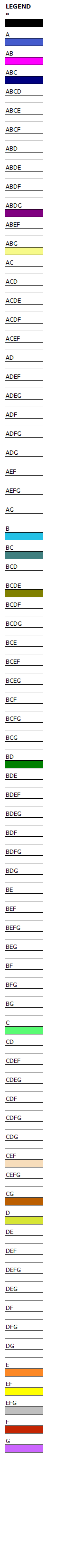


**Figure S5**: Statistical Dispersal-Vicariance Analysis (S-DIVA) of the main dataset (COI, Cytb) of *Erebia oeme* generated with RASP. A – Central and Western Alps, B – Eastern Alps including Slovenia, C – Massif Central, D – Pyrenees, E – Eastern Balkan, F – Western Balkan, G – Julian Alps (Slovenia)

**Figure S6**: Mismatch distribution analysis of the mitochondrial DNA (COI and Cytb) of *Erebia oeme*

**Table S7**: Tajima's D and corresponding p-value of the main dataset (COI, Cytb) of *Erebia oeme* from the different mountain regions and regions. Significant Tajima's D at p-value < 0.05.

| Mountain range | Region | Tajima's D | p-value |
| --- | --- | --- | --- |
| Pyrenees | Pyrenees | -0.718 | 0.472 |
| Massif Central | Massif Central | 0.637 | 0.524 |
| Alps | Alps | -0.932 | 0.352 |
|  | Alps without Slovenia | -0.571 | 0.568 |
|  | Julian Alps (Slovenia) | 0.168 | 0.867 |
|  | Central and Western Alps | 0.174 | 0.862 |
|  | Eastern Alps | -0.168 | 0.866 |
|  | Eastern Alps without Slovenia | 0.497 | 0.619 |
| Non-Balkan | Pyrenees, Massif Central, Alps without Slovenia | -1.360 | 0.174 |
| Balkans | Balkans | 0.414 | 0.679 |
|  | Western Balkan | 0.096 | 0.924 |
|  | Eastern Balkan | **2.162** | 0.031 |

**Table S8**: Infection status of the *Erebia oeme* individuals sorted by populations with *Wolbachia*. A – Strain 1, B – Strain 2, C – Strain 3.

| Sample | Infection Status | Sample | Infection Status | Sample | Infection Status | Sample | Infection Status |
| --- | --- | --- | --- | --- | --- | --- | --- |
| EO001 | A | EO049 | A | EO012 | A | EO071 | A |
| EO002 | No infection | EO050 | A | EO057 | A | EO072 | A |
| EO025 | A | EO051 | A | EO058 | A | EO019 | A |
| EO026 | A | EO052 | A | EO059 | A | EO020 | A |
| EO027 | A | EO053 | A | EO060 | No infection | EO041 | A |
| EO028 | A | EO054 | A | EO061 | A | EO042 | A |
| EO029 | A | EO055 | A | EO062 | No infection | EO043 | A |
| EO030 | A | EO056 | A | EO063 | A | EO044 | A |
| EO031 | A | EO007 | No infection | EO064 | No infection | EO047 | A |
| EO032 | A | EO008 | B | EO101 | A | EO048 | A |
| EO131 | A | EO081 | B | EO102 | A | EO152 | A |
| EO132 | A | EO082 | B | EO103 | A | EO153 | A |
| EO133 | No infection | EO083 | No infection | EO104 | A | EO154 | A |
| EO134 | A | EO084 | A | EO105 | A | EO111 | A |
| EO135 | A | EO085 | No infection | EO106 | A | EO112 | No infection |
| EO136 | A | EO086 | A | EO107 | A | EO113 | No infection |
| EO137 | A | EO087 | No infection | EO108 | A | EO114 | No infection |
| EO138 | A | EO088 | No infection | EO109 | A | EO115 | A |
| EO139 | A | EO009 | A | EO110 | No infection | EO116 | No infection |
| EO140 | A | EO010 | A | EO155 | A | EO117 | A |
| EO121 | A | EO073 | A | EO013 | A | EO118 | A |
| EO122 | A | EO074 | A | EO014 | A | EO119 | A |
| EO123 | A | EO075 | A | EO033 | A | EO120 | No infection |
| EO124 | No infection | EO076 | A | EO034 | A | EO021 | A |
| EO125 | No infection | EO077 | A | EO035 | No infection | EO022 | A |
| EO126 | C | EO078 | A | EO036 | A | EO147 | A |
| EO127 | A | EO079 | A | EO037 | A | EO148 | A |
| EO128 | A | EO080 | A | EO038 | No infection | EO149 | A |
| EO129 | A | EO156 | A | EO039 | No infection | EO150 | A |
| EO130 | A | EO157 | A | EO040 | A | EO151 | A |
| EO003 | A | EO158 | No infection | EO015 | A | EO023 | A |
| EO004 | A | EO159 | No infection | EO016 | A | EO024 | A |
| EO089 | A | EO160 | No infection | EO145 | No infection | EO097 | A |
| EO090 | A | EO161 | A | EO146 | A | EO098 | A |
| EO091 | No infection | EO162 | A | EO017 | A | EO099 | A |
| EO092 | A | EO163 | A | EO018 | A | EO100 | A |
| EO093 | A | EO164 | No infection | EO065 | A | EO141 | A |
| EO094 | A | EO165 | No infection | EO066 | A | EO142 | A |
| EO095 | A | EO166 | No infection | EO067 | A | EO143 | A |
| EO096 | A | EO167 | No infection | EO068 | A | EO144 | A |
| EO005 | A | EO168 | A | EO069 | A |  |  |
| EO006 | A | EO011 | A | EO070 | A |  |  |

**Figure S7**: Maximum Likelihood phylogeny of four detected *Wolbachia* strain sequences.

## References Supplementary

1. Dinca, V., Cuvelier, S., Zakharov, E. V., Hebert, P. D. N. & Vila, R. Biogeography, ecology and conservation of *Erebia oeme* (Hübner) in the Carpathians (Lepidoptera: Nymphalidae: Satyrinae). *Annales de la Société entomologique de France* **46,** 486–498 (2010).

2. Dinca, V. *et al.* High resolution DNA barcode library for European butterflies reveals continental patterns of mitochondrial genetic diversity. *Communications Biology* **4,** 315 (2021).

3. Dapporto, L. *et al.* Integrating three comprehensive data sets shows that mitochondrial DNA variation is linked to species traits and paleogeographic events in European butterflies. *Mol Ecol Resour* **19,** 1623–1636 (2019).

4. Huemer, P. & Hebert, P. D. N. DNA-Barcoding der Schmetterlinge (Lepidoptera) Vorarlbergs (Österreich)-Erkenntnisse und Rückschlüsse. *Inatura Forschung online* **15,** 1–36 (2015).

5. Hausmann, A. *et al.* Now DNA-barcoded: the butterflies and larger moths of Germany. *Spixiana* **34,** 47–58 (2011).

6. Mutanen, M. *et al.* Species-level para-and polyphyly in DNA barcode gene trees: strong operational bias in European Lepidoptera. *Systematic Biology* **65,** 1024–1040 (2016).

7. Litman, J. *et al.* A DNA barcode reference library for Swiss butterflies and forester moths as a tool for species identification, systematics and conservation. *PloS One* **13,** e0208639 (2018).

8. Peña, C., Witthauer, H., Klečková, I., Fric, Z. & Wahlberg, N. Adaptive radiations in butterflies: evolutionary history of the genus *Erebia* (Nymphalidae: Satyrinae). *Biological Journal of the Linnean Society* **116,** 449–467 (2015).

9. Carnicer, J., Stefanescu, Constanta, Vlad, Font, X. & Peñuelas, J. A unified framework for diversity gradients: the adaptive trait continuum. *Global Ecology and Biogeography* **22,** 6–18 (2013).

10. Dinca, V. *et al.* DNA barcode reference library for Iberian butterflies enables a continental-scale preview of potential cryptic diversity. *Scientific Reports* **5,** 12395 (2015).

11. Johanson, K. A. & Malm, T. Testing the monophyly of Calocidae (Insecta: Trichoptera) based on multiple molecular data. *Molecular Phylogenetics and Evolution* **54,** 535–541 (2010).

12. Malm, T. & Nyman, T. Phylogeny of the symphytan grade of Hymenoptera: new pieces into the old jigsaw (fly) puzzle. *Cladistics* **31,** 1–17 (2015).

13. Cho, S. *et al.* A highly conserved nuclear gene for low-level phylogenetics: elongation factor-1 alpha recovers morphology-based tree for heliothine moths. *Molecular Biology and Evolution* **12,** 650–656 (1995).

14. Nyman, T., Zinovjev, A. G., Vikberg, V. & Farrell, B. D. Molecular phylogeny of the sawfly subfamily Nematinae (Hymenoptera: Tenthredinidae). *Systematic Entomology* **31,** 569–583 (2006).

15. Wahlberg, N. & Wheat, C. W. Genomic outposts serve the phylogenomic pioneers: designing novel nuclear markers for genomic DNA extractions of Lepidoptera. *Systematic Biology* **57,** 231–242 (2008).

16. Wahlberg, N., Peña, C., Ahola, M., Wheat, C. W. & Rota, J. PCR primers for 30 novel gene regions in the nuclear genomes of Lepidoptera. *ZooKeys,* 129 (2016).

17. Brower, A. V. & DeSalle, R. Patterns of mitochondrial versus nuclear DNA sequence divergence among nymphalid butterflies: the utility of wingless as a source of characters for phylogenetic inference. *Insect Molecular Biology* **7,** 73–82 (1998).
